# Genetically determined tobacco and alcohol use and risk of atrial fibrillation

**DOI:** 10.1186/s12920-021-00915-0

**Published:** 2021-03-09

**Authors:** Yunlong Lu, Yan Guo, Hefeng Lin, Zhen Wang, Liangrong Zheng

**Affiliations:** 1 Department of Cardiology, The First Affiliated Hospital, School of Medicine, Zhejiang University, Hangzhou, 310003 Zhejiang China; 2 Zhejiang University, Hangzhou, 310003 Zhejiang China

**Keywords:** Smoking, Alcohol, Mendelian randomization, Atrial fibrillation

## Abstract

**Background:**

The causality between the use of alcohol and cigarettes and atrial fibrillation (AF) remains controversial. We conducted a Mendelian randomization (MR) study to evaluate the association of genetic variants related to tobacco and alcohol use with AF.

**Methods:**

Single nucleotide polymorphisms (SNPs) related to smoking initiation (N = 374), age at initiation of regular smoking (N = 10), cigarettes per day (N = 55), and smoking cessation (N = 24) were derived from a genome-wide association studies (GWAS) of tobacco use (N = 1.2 million individuals). SNPs related to heavy alcohol use (N = 6) were derived from a GWAS of UK biobank (N = 125,249 individuals). The genetically matching instrumented variables were obtained from the GWAS of AF (N = 588,190 individuals). The estimates between tobacco and alcohol use and AF were combined by inverse-variance weighted (IVW), simple median, weighted median, MR-robust adjusted profile score method, MR-PRESSO, and multivariable MR.

**Results:**

A total of 65,446 AF patients and 522,744 referents were included. In the IVW analysis, the odds ratio per one-unit increase of smoking initiation was 1.11 (95% CI, 1.06–1.16; *P* = 3.35 × 10^−6^) for AF. Genetically predicted age at initiation of regular smoking, cigarettes per day and smoking cessation were not associated with AF. The IVW estimate showed that heavy alcohol consumption increased AF risk (OR, 1.11; 95% CI, 1.04–1.18; *P* = 0.001). The results were consistent in complementary analyses and multivariable MR.

**Conclusion:**

Our MR study indicated that regular smoking was associated with increased risk of AF, no matter the age at initiation of regular smoking, or the number of cigarettes smoked per day. Genetically predicted heavy alcohol consumption increased the risk of AF.

## Background

Cigarette smoking and alcohol intake are potential modifiable risk factors for atrial fibrillation (AF), which is the most common sustained arrhythmia and associated with an increased risk of stroke, heart failure and even death [1]. Several observational studies have suggested that smoking is associated with incident AF [2–5]. Meanwhile, whether the number of cigarettes smoked and smoking cessation paly a causal role in the increased AF risk is controversial [2, 3, 6]. While a series of previous studies have shown that low to moderate alcohol intake did not demonstrate a significant association with AF [7–9], there was evidence to prove an elevated risk of AF with high alcohol intake [10, 11]. Nevertheless, observational studies for causal inference were susceptible to confounding factors and reverse causal inference, which might result in an unreliable conclusion [12].

Mendelian randomization (MR) analysis is a method using instrumental variables associated with exposure to infer causality and interactions in observational studies [13], which could diminish the confounders of observed associations between exposures and outcomes [14]. Hence, to examine the potential role of smoking and drinking for AF risk, we conducted a two-sample MR analysis, using single nucleotide polymorphisms (SNPs) associated with tobacco and alcohol use as instrumental variables.

## Methods

### Study design

The diagram of the MR assumptions underpinning a MR analysis of the association of tobacco and alcohol use on AF was shown in the Additional file 1: Fig. 1 . Tobacco and alcohol use associated instrumental variables were looked-up in the outcome GWAS by querying the matched SNPs. While SNPs were not available in the outcome GWAS, proxies were used in the European population genotype data originated from Phase 3 (Version 5) of the 1000 Genomes Project (linkage disequilibrium [LD] *r*^*2*^ > 0.8; identified using online tool SNiPa, available at: http://snipa.helmholtz-muenchen.de/snipa3/).

### Data source

In this study, publicly available data were used to conduct the MR analyses. All participants of the original studies included in the GWASs had provided informed written consent. The data of tobacco and heavy alcohol use were extracted from a large-scale genome-wide association studies with smoking initiation, cigarettes per day, smoking cessation, age at initiation of regular smoking and heavy alcohol intake (Additional file 1: Table 1) [15, 16]. Genetic instrumental variables for the exposure were selected at the genome-wide significance level (*P* < 5 × 10^−8^). Specially, smoking phenotypes associated instrumental variables were defined as a 1-Mb region nearby the top *P*-value.

### Tobacco and alcohol use

Smoking initiation was defined ever being a regular smoker in the life (current or former). The GWAS on smoking initiation identified 378 single nucleotide polymorphisms (SNPs), explaining 2.3% of the heritability in up to 1,232,091 individuals. While 10 SNPs were unavailable in the AF database, 6 proxy SNPs in LD (*r*^*2*^ ≥ 0.8) with the specified SNPs were used. Finally, 374 out of 378 SNPs were included in the analyses (Additional file 1: Table 2).

The GWAS identified 10 SNPs associated with age at initiation of regular smoking, explaining 0.2% of the heritability in up to 341,427 individuals, 1 SNPs (rs12611472) was not found in the AF database and no appropriate proxy SNP was available. Thus, 9 SNPs were selected as instrumental variables (Additional file 1: Table 3).

The 55 SNPs related to cigarettes per day (current smoker or former smoker) in 337,334 individuals were estimated to explain 1.1% of the variation in cigarettes per day. There was 1 SNP (rs4886550) not found in the AF database and we failed to match the appropriate proxy SNP. Therefore, 54 SNPs associated with cigarettes per day were used as instrumental variables in the MR analyses (Additional file 1: Table 4).

The all 24 SNPs associated with smoking cessation (current smoker vs. former smoker) were identified as instrumental variables in 547,219 individuals, which explained 0.1% of the variation in smoking cessation (Additional file 1: Table 5).

The GWAS on heavy alcohol drinking (defined as > 35 U/week for women and > 50 U/week for men, see the Supplementary Methods for more detail) identified 6 SNPs, explaining 2.0% of the variance in heavy alcohol use, in up to 21,967 cases and 103,282 controls of European ancestry from UK Biobank. All 6 SNPs were included in the analyses (Additional file 1: Table 6).

We used *F* statistics to estimate the strength of genetic instruments [17]. For the instrument SNPs selected in this study, *F* statistics ranged from 28 to 1343, above the recommended threshold of *F* > 10 in MR analysis [17]. We further clumped these variants to an LD threshold of *r*^*2*^ < 0.1 in European populations using LDlink [18], retaining SNPs with the lowest *P* value (Additional file 1: Fig. 2).

### Atrial fibrillation

Summary statistics for the associations of tobacco and alcohol use-SNP with AF were obtained from the genome-wide association studies (GWAS) of 4 consortiums and databases, including AFGen consortium (23,685 AF cases, 148,193 references), Broad AF study (17,517 AF cases, 10,987 references), UK Biobank (16,064 AF case, 334,935 references), and Biobank Japan (8180 AF cases, 28,612 references) (Additional file 1: Tables 1 and 7) [19]. AF cases include participants with paroxysmal or permanent AF, or atrial flutter, shown in the each study respectively. The 9/10th revision of International Classification of Diseases (ICD9/10) codes was used to define AF patients in the UK Biobank with at least one of following codes: non-cancer illness code, self-reported (1471, 1483); operation code (1524); diagnoses – main/secondary ICD10 (I48, I48.0-4, I48.9); underlying (primary/secondary) cause of death: ICD10 (I48, I48.0-4, I48.9); diagnoses—main/secondary ICD9 (4273); operative procedures—main/secondary OPCS (K57.1, K62.1-4) [19]. We have conducted sensitivity analyses restricted to individuals of European ancestry (60,620 atrial fibrillation cases; 970,216 controls) from six contributing studies (The Nord-Trøndelag Health Study, deCODE, the Michigan Genomics Initiative, DiscovEHR, UK Biobank, and the AFGen Consortium) [20], to minimize the influence of population stratification caused by ancestral confounding.

### Statistical analysis

A fixed-effects inverse-variance weighted (IVW) meta-analysis was used to combine the estimates. Complementary analyses using the simple median, weighted median, MR-robust adjusted profile score (MR-RAPS, an MR method for correcting for horizontal pleiotropy using robust adjusted profile scores [21]), and MR-pleiotropy residual sum and outlier method (MR-PRESSO, an approach to detect and correct for horizontal pleiotropic outliers in IVW [22]) methods were also adopted. Heterogeneity statistics were conducted by means of IVW methods. The result estimate of IVW method was considered credible if there was no directional pleiotropy (*P* for MR-Egger intercept > 0.05). In addition, there was a genetic correction between smoking and alcohol consumption (r_g_ = 0.34) [15]. To intervene on interplay of smoking and alcohol intake to causal estimates, we conducted the multivariable MR [23], using publicly available summarized data for genetic association of instruments with smoking and alcohol use from the GWAS and Sequencing Consortium of Alcohol and Nicotine use [15]. For each exposure, we use associated SNPs (372 for smoking initiation, 9 for age at initiation of regular smoking, 54 for cigarettes per day, 24 for smoking cessation, and 6 for heavy alcohol drinking, respectively) to illustrate the potential confounding by alcohol consumption in smoking-AF risk or smoking in alcohol-AF risk. Statistical significance for associations with AF was set at *P* < 0.05/5 = 0.01 for 5 exposures (4 smoking phenotypes and heavy alcohol use) corrected for multiple comparisons using the Bonferroni method. A two-sided *P* value of < 0.05 was considered suggestive for significance. MR analyses were conducted using the TwoSampleMR and MR-pleiotropy residual sum and outlier R packages. All analyses were conducted in R version 3.6.1.

## Results

The analytic sample included 65,446 atrial fibrillation patients (84.2% European, 12.5% Japanese, 2.0% African American, 0.9% Brazilian and 0.4% Hispanic populations) and 522,744 referents from case–control and population-based studies from 4 large-scale consortiums and databases (Additional file 1: Tables 1 and 7).

### Tobacco use and AF

**Fig. 1:**
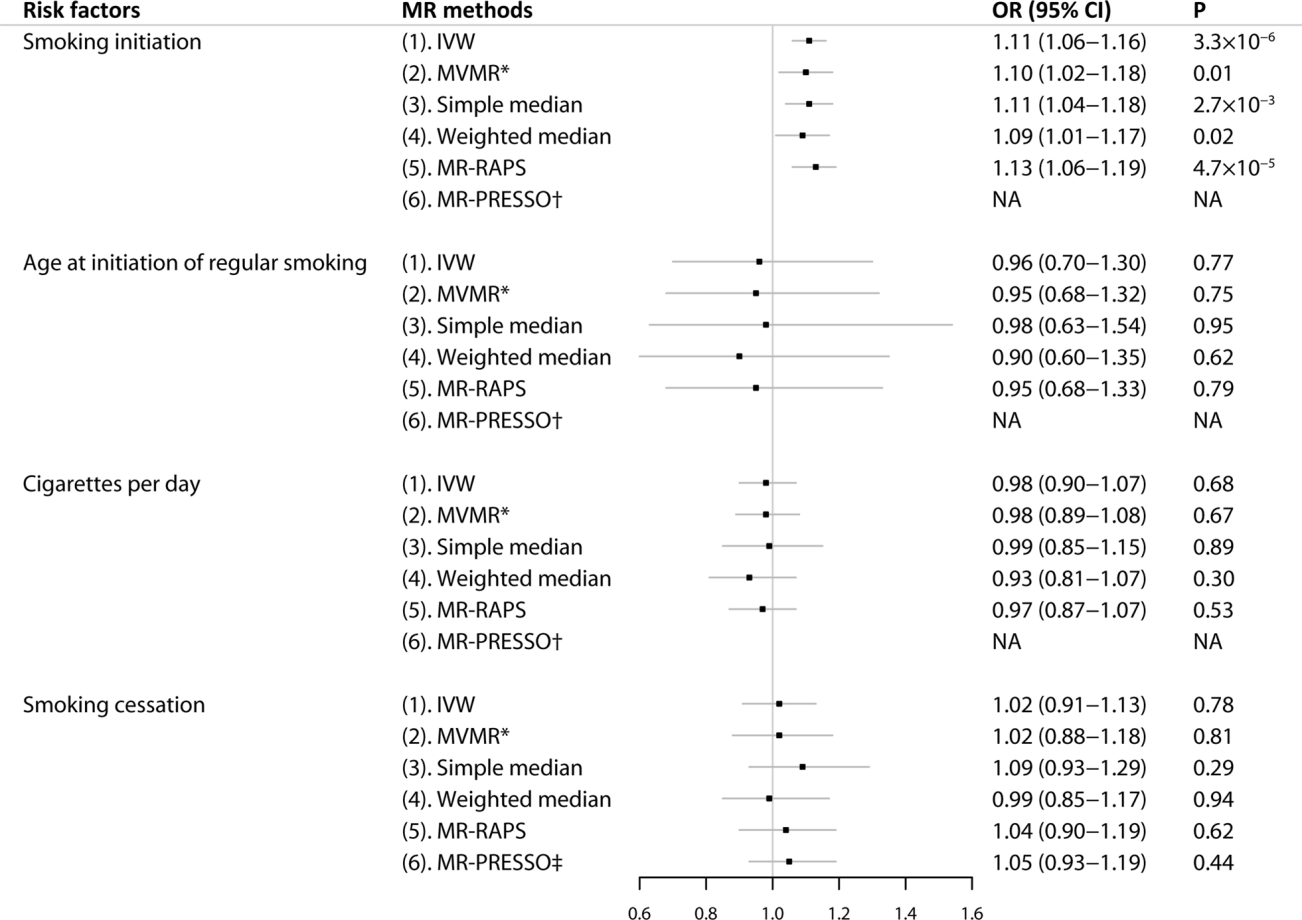
Mendelian randomization association of smoking initiation, age at initiation of regular smoking, cigarettes per day and smoking cessation on atrial fibrillation using genetic instrument variables. Odds ratios are scaled to per one-unit increase in genetically predicted smoking initiation (about 10–12% increase in the probability of being a regular smoker), age at initiation of regular smoking (about additional 0.31–0.50 years), cigarettes per day (about 2–3 additional cigarettes daily) and smoking cessation (about 3–5% increase in the probability of being a current smoker). *Adjusted for alcohol drinking. ^†^No outlier detected. ^‡^MR-PRESSO instrumental variable outlier detected: rs3025327. CI, confidence interval; IVW, the inverse-variance weighted method; MR, Mendelian randomization; MR-RAPS, MR-robust adjusted profile scores; MR-PRESSO, MR-pleiotropy residual sum and outlier; MVMR, multivariable Mendelian randomization; OR, odds ratio

Genetically predicted smoking initiation was associated with a higher risk of AF in the standard IVW analysis, with an OR of 1.11 (95% CI, 1.06–1.16; *P* = 3.35 × 10^–6^) per one-unit increase (about 10–12% increase in the probability of being a regular smoker, see the Supplementary Methods for more detail) of smoking initiation without detected pleiotropy bias (MR-Egger intercept, -0.0018; *P* = 0.44) (Fig. 1). The association between genetically instrumented smoking initiation and AF was in line with complementary analyses using the multivariable MR adjusted for alcohol intake (OR, 1.10; 95% CI, 1.02–1.18), the simple median methods (OR, 1.11; 95% CI, 1.04–1.18), weighted median method (OR, 1.09; 95% CI, 1.01–1.17), and MR-RAPS (OR, 1.13; 95% CI, 1.06–1.19) (Fig. 1). There was a moderate heterogeneity between Mendelian randomization estimates of different SNPs (*I*^*2*^ = 43.6%).

Genetically predicted age at initiation of regular smoking, cigarettes per day and smoking cessation were not associated with AF in any MR analyses (Fig. 1). No indication of directional pleiotropy was found in the MR-Egger intercept analysis (*P* = 0.54 for age at initiation of regular smoking, *P* = 0.21 for cigarettes per day, and *P* = 0.32 for smoking cessation, respectively), and there was moderate heterogeneity among SNPs (*I*^*2*^ = 46.1%) of smoking cessation. The IVW showed that associations of genetically predicted tobacco use with AF were consistent using an LD of *r*^*2*^ < 0.1 and in European-ancestry individuals (Additional file 1: Figs. 2 and 3).

### Alcohol use and AF

**Fig. 2:**
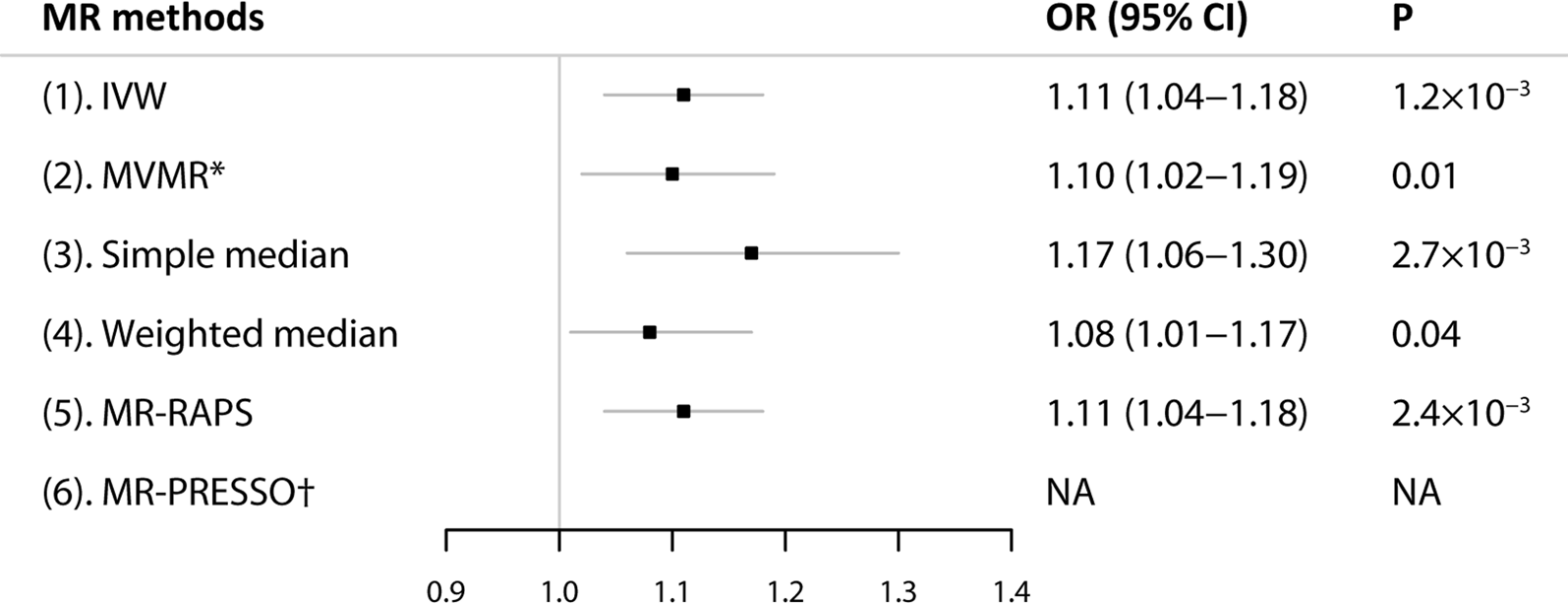
Mendelian randomization association between genetically predicted heavy alcohol use and atrial fibrillation. Odds ratio is scaled to per one-unit increase in genetically predicted heavy alcohol use. *Adjusted for smoking. †No outlier detected. CI, confidence interval; IVW, the inverse-variance weighted method; MR, Mendelian randomization; MR-RAPS, MR-robust adjusted profile scores; MR-PRESSO, MR-pleiotropy residual sum and outlier; MVMR, multivariable Mendelian randomization; OR, odds ratio

The IVW estimate showed that genetically instrumented heavy alcohol consumption increased AF risk (OR, 1.11; 95% CI, 1.04–1.18; *P* = 0.001) (Fig. 2). The association between heavy alcohol use and AF was consistent in complementary analyses using the multivariable MR adjusted for smoking (OR, 1.10; 95% CI, 1.02–1.19), the simple median methods (OR, 1.17; 95% CI, 1.06–1.30), weighted median method (OR, 1.08; 95% CI, 1.01–1.17), and MR-RAPS (OR, 1.11; 95% CI, 1.04–1.18) (Fig. 2). There was no evidence of directional pleiotropy (MR-Egger intercept, 0.0072; *P* = 0.30), and a low heterogeneity between heavy alcohol use and AF (*I*^*2*^ = 22.5%). The significant association between heavy alcohol use and AF was stable when restricting the analysis to an LD of *r*^*2*^ < 0.1 and individuals of European ancestry (Additional file 1: Figs. 2 and 3).

## Discussion

This MR analyses indicated that regular smoking (current or former) was associated with increased risk of AF, no matter the age at initiation of regular smoking, or the number of cigarettes smoked per day. Genetically predicted heavy alcohol consumption increased the risk of AF.

Our findings are in line with a recent meta-analysis of 29 prospective studies demonstrating that current, former and ever smoking were associated with a 32%, 9% and 21% increase of AF risk compared with the never smoking [24]. We found that there was no benefit favoring smoking cessation for AF and no association between cigarettes per day and increased AF risk. While Aune et al. [24] reported an increased rate of AF (relative risks [RR], 1.33; 95% CI, 1.14–1.56) in current smokers compared with the former smokers although there was a high heterogeneity (*I*^*2*^ = 78%) among studies. In the Atherosclerosis Risk in Communities (ARIC) study, the incidence of AF was lower in people who quitted smoking as compared with current smokers, although statistically nonsignificant (hazard ratio [HR], 0.88; 95% CI, 0.65–1.17) [25]. What’s more, Aune et al. [24] showed there was a dose-dependent association between the number of cigarettes smoked and pack-years and increased AF risk, per 10 cigarettes per day was associated with a 14% increased risk of AF. With 3 studies [2, 3, 5] included in the meta-analysis of cigarettes per day and AF, 2 studies [3, 5] only adjusted one or two confounders (age and sex), which might give rise to be overestimated.

On the one hand, smoking regularly can lead to myocardial ischemia by increasing systemic catecholamine, reducing oxygen-carrying capacity and promoting coronary artery contraction [26]. On the other hand, smoking regularly also promotes atherosclerosis through effects on endothelial function, oxidative stress and thrombosis [27]. In view of that smoking has complex effects on cardiovascular diseases, the evidence of whether smoking is an independent risk factor was confusing. Some studies showed that smoking increased the risk of myocardial infarction [28], heart failure [29], diabetes [30], chronic obstructive pulmonary disease [31], which in turn contributed to the onset of AF [32]. In addition, never smokers live a healthier lifestyle normally [33], making it more difficult to figure out the actual impact of smoking on AF. Our MR estimates were less affected by bias with tobacco and alcohol use associated SNPs. Furthermore, there was no pleiotropy between exposure associated SNPs and AF by using MR-Egger intercept, indicating a more reliable conclusion of the MR analysis.

Larsson et al. [10] performed a large prospective study cohort including 79,019 participators free from AF at the baseline, and with information of alcohol consumption. After a follow-up period of 12 years, 7,245 incident AF cases were identified in the study. Results showed that, compared with current alcohol drinkers of less than 1 drink/week (standard drinks = 12 g alcohol), alcohol intake of 15 to 21 drinks/week and > 21 drinks/week had a higher risk of AF (RR, 1.14; 95%CI, 0.51–0.69, and RR, 1,39; 95%CI, 1.22–1.58, respectively). No significant differences in risk of AF were observed in alcohol intake of 1 to 6 and 7 to 14 drinks/week groups. Furthermore, a meta-analysis including 7 prospective studies with 12,554 AF patients was conducted to assess the association of alcohol intake and risk of AF [10]. The meta-analysis suggested that even alcohol intake of 1 drink/day was associated with a statistically significant 8% increased risk of AF.

In a recent prospective, open-label, randomized, controlled trial conducted by Voskoboinik et al. [11] using data from six hospitals in Australia showed that alcohol abstinence reduced arrhythmia recurrences in regular drinkers with AF. Of 140 patients who consumed more than 10 standard drinks (1 standard drink ≈12 g alcohol) per week and who had paroxysmal or persistent atrial fibrillation in sinus rhythm at baseline, 70 were randomly assigned to the abstinence group and 70 to the control group. At 6 months, compared with the control group, the abstinence group had a lower rate of AF recurrence (53% vs 73%) and a longer period before recurrence of AF (HR, 0.55; 95% CI, 0.36–0.84; *P* = 0.005).

### Limitations

Our study is the first to use MR analysis to explore the relationship between tobacco and alcohol use and atrial fibrillation. One of the strengths of this study is the large sample size of more than half a million individuals and up to 65,446 AF cases; the other is biases that potentially exist in conventional observational studies could be reduced. However, our MR analyses were subject to some limitations. First, the study lacked complete data such as sex, we therefore cannot evaluate the association between alcohol intake and AF in different genders, considering some observational studies reporting an association in males and AF at high levels of alcohol intake, but not females [34–36]. Second, pleiotropy may affect the results. Despite the lack of indication of directional pleiotropy in the analysis of tobacco and alcohol use and AF, we cannot exclude that the association is mediated through other causal pathways, especially considering instability of the alcohol intake in genetic predisposition [37]. Third, with a majority of the participants of European descent, it might limit the scope of our finding in other ancestral groups. Fourth, we also cannot entirely eliminate the influence of population stratification (confounding by multiple ancestry). However, because around 91% individuals were European ancestry, this bias was limit. In addition, the analyses of genetic associations with atrial fibrillation in European ancestry only yielded consistent results. Fifth, there was somehow a sample overlap, though quite small, in the studies of AF database. Finally, due to the assumption of linear relationship between exposure and outcome in MR design [38], a potential nonlinear association of smoking and heavy alcohol drinking with AF could not be evaluated. However, with large sample size included and the consistent results in sensitivity analyses, the influence of bias owing to binary outcome was minimized in our study.

## Conclusion

In conclusion, our MR study indicated that regular smoking might be associated with increased risk of AF, no matter the age at initiation of regular smoking, or the number of cigarettes smoked per day. The result also showed that heavy alcohol intake was linked to an increased risk of AF.

## Supplementary Information

**Additional file 1.** Supplementary methods. **Table S1**. Descriptive information of the studies and datasets included in the analyses. **Table S2**. Characteristics of the genetic variants associated with smoking initiation. **Table S3**. Characteristics of the genetic variants associated with age at initiation of regular smoking. **Table S4**. Characteristics of the genetic variants associated with cigarettes per day. **Table S5**. Characteristics of the genetic variants associated with smoking cessation. **Table S6**. Characteristics of the genetic variants associated with heavy alcohol drinking. **Table S7**. Summary of atrial fibrillation cases and referents by ancestry. **Figure S1**. Diagram of the Mendelian randomization assumptions underpinning a Mendelian randomization analysis of the association of smoking and alcohol use on atrial fibrillation. **Figure S2**. Mendelian randomization association of genetically predicted smoking and heavy alcohol use with atrial fibrillation, using a linkage disequilibrium threshold of r2<0.1. **Figure S3**. Mendelian randomization association of genetically predicted smoking and heavy alcohol use with atrial fibrillation in multiple ancestry and European ancestry

## Data Availability

This study used publicly available data of genome-wide association study. The summary statistics for tobacco and alcohol use are reported by Refs. 15–16. The summary statistics for atrial fibrillation risk are derived from the 2018 AF HRC GWAS, which can be accessed at http://kp4cd.org/datasets/mi. No permissions are required to access these data.

## Abbreviations

AF: Atrial fibrillation
ARIC: Atherosclerosis Risk in Communities
GWAS: Genome-wide association studies
ICD: International Classification of Diseases
CI: Confidence interval
IVW: The inverse-variance weighted method
LD: Linkage disequilibrium
MR: Mendelian randomization
MR-RAPS: MR-robust adjusted profile scores
MR-PRESSO: MR-pleiotropy residual sum and outlier
OR: Odds ratio
RR: Relative risks
SNPs: Single nucleotide polymorphisms
