# Supplementary material for: Genetically determined tobacco and alcohol use and risk of atrial fibrillation
Source: BMC Med Genomics. 2021 Mar 9;14:73. doi: 10.1186/s12920-021-00915-0 (PMC7944892; doi:10.1186/s12920-021-00915-0)
Supplement: Supplementary file 1 — Additional file 1. Supplementary methods. Table S1. Descriptive information of the studies and datasets included in the analyses. Table S2. Characteristics of the genetic variants associated with smoking initiation. Table S3. Characteristics of the genetic variants associated with age at initiation of regular smoking. Table S4. Characteristics of the genetic variants associated with cigarettes per day. Table S5. Characteristics of the genetic variants associated with smoking cessation. Table S6. Characteristics of the genetic variants associated with heavy alcohol drinking. Table S7. Summary of atrial fibrillation cases and referents by ancestry. Figure S1. Diagram of the Mendelian randomization assumptions underpinning a Mendelian randomization analysis of the association of smoking and alcohol use on atrial fibrillation. Figure S2. Mendelian randomization association of genetically predicted smoking and heavy alcohol use with atrial fibrillation, using a linkage disequilibrium threshold of r2<0.1. Figure S3. Mendelian randomization association of genetically predicted smoking and heavy alcohol use with atrial fibrillation in multiple ancestry and European ancestry [file 12920_2021_915_MOESM1_ESM.docx]

# Supplemental Material

Genetically determined tobacco and alcohol use and risk of atrial fibrillation

Yunlong Lu, Yan Guo, Hefeng Lin, Zhen Wang, Liangrong Zheng

# ****Supplementary Methods****

**1. Phenotype definitions**

**1.1 Smoking Initiation**

This is a binary phenotype. Any participant reporting ever being a regular smoker in their life (current or former) were coded “2”, while any participant who reported never being a regular smoker in their life were coded “1”.

**1.2 Cigarettes per Day**

Defined as the average number of cigarettes smoked per day, either as a current smoker or former smoker, and whether self-rolled or manufactured are smoked. Individuals who either never smoked, or for whom there is no available data (e.g., someone was a former smoker, but for whom former smoking was never assessed) were set to missing.

**1.3 Smoking Cessation**

This is a binary phenotype with current smokers coded as “2” and former smokers coded as “1”, and never smokers are coded as missing.

**1.4 heavy alcohol consumption**

UK alcohol units applied to each alcoholic drink in UK Biobank cohort

The number of units in each type of alcoholic drink was standardised using drink measure (provided by UK) and alcohol by volume data from various sources. Units were determined by multiplying the volume of the drink (in millilitres) by its percentage alcohol by volume, and dividing the outcome by 1000.

The units assigned to each drink (Beer/cider = 2.6; White wine = 1.5; Red wine = 1.5; Fortified wine = 1.1; Spirits = 1; Other =1.5) were multiplied by the self-reported number of specific drinks consumed per week or per month and then summed across all types of drinks. Where information was only available on alcohol consumed per month, the summed data was multiplied by 12 (for months in a year) and then divided by 52 to obtain a measure comparable with weekly alcohol intake.[^1^](#_ENREF_1)

**2. Polygenic scoring results**
In the GWAS of genetically determined tobacco use[^2^](#_ENREF_2), two independent prediction samples, the National Longitudinal Study of Adolescent to Adult Health (Add Health)[^3^](#_ENREF_3) and the Health and Retirement Study (HRS)[^4^](#_ENREF_4) datasets, were selected to assess the predictive ability for genetically predicted smoking phenotypes. Add Health and HRS are representatives of their birth cohorts in the United States and represent exposures to different tobacco policy environments.

The sizes of one standard-deviation increase associated with four smoking phenotypes are described below.

1) For smoking initiation, a one standard-deviation increase was associated with a 12% increase in the probability of being a regular smoker in Add Health; the comparable increase is 10% in the HRS.

2) For age at initiation of regular smoking, a one standard-deviation increase is associated with an additional 0.31 years in the age at smoking initiation in Add Health and an additional 0.50 years in the HRS.

3) For cigarettes per day, a one standard-deviation increase is associated with about two additional cigarettes daily in Add Health and about three additional cigarettes in the HRS.

4) For smoking cessation, a one standard-deviation increase in the score was associated with a 5% increase in the probability of being a current (versus former) smoker in Add Health and a 3% increase in the HRS.

## Supplementary Table 1. Descriptive information of the studies and datasets included in the analyses

| **Exposure/Outcome** | **Participants** | **Consortium or cohort study** | **Web source** |
| --- | --- | --- | --- |
| Smoking initiation | 123,2091 individuals of European ancestry | Add Health, ALSPAC, ARIC, BBJ, BEAGESS, BLTS, CADD, CPGEND, COPDGene, deCODE, EGCUT, MERGE, Finntwin & NAG-FIN, FHS, GERA, GfG, HUNT, HRS, MCTFR, MESA, METSIM, NESCOG, NHS, NHS2, HPFS, NINDS SiGN, NTR, OZALC, SardiNIA, UK Biobank, WHI | <https://conservancy.umn.edu/handle/11299/201564> |
| Age at initiation of regular smoking | 341,427 individuals of European ancestry |  |  |
| Cigarettes per day | 337,334 individuals of European ancestry |  |  |
| Smoking cessation | 547,219 individuals of European ancestry |  |  |
| heavy alcohol drinking | 21,967 cases and 103,282 controls of European ancestry | UK Biobank | https://www.ncbi.nlm.nih.gov/pmc/articles/PMC6962045/#!po=8.59375 |
| Atrial fibrillation | 65,446 atrial fibrillation patients (84.2% European, 12.5% Japanese, 2% African American, 0.9% Brazilian and 0.4% Hispanic populations) and 522744 referents | AFGen consortium (23685 AF cases, 148193 references), Broad AF study (17517 AF cases, 10987 references), UK Biobank (16064 AF case, 334935 references), Biobank Japan (8180 AF cases, 28612 references) (AGES, BEAT-AF, BioMe, AFCT, AFLMU, Australian Familial AF Study, BioVU, Danish AF Study, Duke, EAST AFNET4, GENAF, GGAF, GRADE, GerMIFS, German Heart Center Controls, HVH, Hopkins, Intermountain, MGH AF, MGH DOFEGEN, MGH Stroke, MPP AF, MPP Echo, Penn, TCAI, UCSF, UMass, VAFAR, CCAF, Corogene, FHS, GS:SFHS, LURIC, MDCS, MGH CAMP, SiGN Group1, SiGN Group2, SiGN Group4, SiGN Group5, SiGN Group6, SiGN Group7, SiGN Group8, SiGN Group9, Vanderbilt AF Registry, WTCCC2 Munich, ARIC, CHS , MESA, PIVUS, PROSPER, TWINGENE, ULSAM, WGHS, ANGES, EGCUT 370, EGCUT Omni, FINCAVAS, PHB (MEGA), PHB (MEGA Ex), PHB (MEG), PREVEND, RS1, RS2, RS3, SHIP, UK Biobank, Broad AF Study, CHS, SPHFC, Broad AF Study , Incor Warfarin Study, BBJ) | <http://www.broadcvdi.org/informational/data> |

## Supplementary Table 2. Characteristics of the genetic variants associated with smoking initiation

| **SNP** | **EA** | **NA** | **EAF** | **Chr** | **Position** | **Gene** | **Beta*** | **Se** | **P-value** |
| --- | --- | --- | --- | --- | --- | --- | --- | --- | --- |
| rs12130857 | A | G | 0.32 | 1 | 7791461 | Intron:CAMTA1 | -0.018 | 0.003 | 3.7E-11 |
| rs301807 | G | A | 0.57 | 1 | 8484823 | Intron:LOC102724552\|RERE | 0.018 | 0.003 | 2.5E-12 |
| rs3820277 | T | G | 0.53 | 1 | 18436657 | Intron:IGSF21 | -0.019 | 0.003 | 1.6E-13 |
| rs1889571 | G | T | 0.13 | 1 | 32195819 | Intron:ADGRB2 | 0.022 | 0.004 | 4.2E-09 |
| rs10914684 | A | G | 0.32 | 1 | 33795572 | Intron:PHC2 | -0.016 | 0.003 | 6.3E-09 |
| rs2637869 | A | G | 0.30 | 1 | 38757237 | Intergenic | 0.018 | 0.003 | 6.5E-11 |
| rs12755632 | G | A | 0.32 | 1 | 41776623 | Intergenic | -0.015 | 0.003 | 1.9E-08 |
| rs951740 | A | G | 0.63 | 1 | 44011737 | Intron:PTPRF | 0.030 | 0.003 | 3.8E-29 |
| rs925524 | G | A | 0.71 | 1 | 46496709 | Synonymous:MAST2 | 0.016 | 0.003 | 2.9E-08 |
| rs12022778 | C | A | 0.20 | 1 | 50603995 | Intron:ELAVL4 | 0.027 | 0.003 | 3.2E-17 |
| rs11587399 | T | A | 0.22 | 1 | 50861071 | Intergenic | -0.018 | 0.003 | 7.3E-09 |
| rs4912332 | T | C | 0.49 | 1 | 58815243 | Intergenic | 0.014 | 0.003 | 2.9E-08 |
| rs1937443 | G | C | 0.56 | 1 | 66469643 | Intron:PDE4B | 0.020 | 0.003 | 1.8E-15 |
| rs1022528 | A | G | 0.34 | 1 | 71490122 | Intron:PTGER3 | 0.017 | 0.003 | 8.5E-11 |
| rs12740789 | A | G | 0.18 | 1 | 72752073 | Intergenic | -0.028 | 0.003 | 1.2E-17 |
| rs80054503 | C | T | 0.12 | 1 | 72900406 | Intergenic | -0.024 | 0.004 | 3.1E-09 |
| rs10789369 | G | A | 0.62 | 1 | 73824909 | Intergenic | -0.023 | 0.003 | 3.4E-19 |
| rs1514176 | A | G | 0.58 | 1 | 74991596 | Intron:FPGT-TNNI3K\|TNNI3K | -0.019 | 0.003 | 7.7E-14 |
| rs10873871 | G | A | 0.21 | 1 | 76689019 | Intron:ST6GALNAC3 | 0.017 | 0.003 | 2.8E-08 |
| rs11162019 | T | C | 0.36 | 1 | 87913176 | Intergenic | -0.015 | 0.003 | 5.1E-09 |
| rs1008078 | T | C | 0.40 | 1 | 91189731 | Intergenic | 0.023 | 0.003 | 1.6E-18 |
| rs1935571 | G | T | 0.48 | 1 | 96414335 | Intergenic | -0.016 | 0.003 | 7.0E-10 |
| rs12027999 | C | T | 0.12 | 1 | 154206358 | Intron:UBAP2L | -0.024 | 0.004 | 5.3E-10 |
| rs45444697 | G | C | 0.21 | 1 | 155034632 | Intron:ADAM15\|DCST1-AS1 | 0.020 | 0.003 | 2.7E-10 |
| rs2901785 | A | G | 0.45 | 1 | 174104743 | Intron:LOC102724601 | -0.017 | 0.003 | 1.5E-11 |
| rs147052174 | T | G | 0.02 | 1 | 179783167 | Nonsynonymous:FAM163A | 0.062 | 0.010 | 2.3E-10 |
| rs35656245 | A | G | 0.28 | 1 | 190957480 | Intergenic | 0.016 | 0.003 | 2.2E-08 |
| rs12739243 | C | T | 0.22 | 1 | 210302043 | Intron:SYT14 | -0.021 | 0.003 | 4.5E-12 |
| rs12563365 | A | G | 0.56 | 1 | 236872829 | Intron:ACTN2 | 0.017 | 0.003 | 1.1E-10 |
| rs876793 | C | T | 0.35 | 1 | 237852083 | Intron:RYR2 | -0.018 | 0.003 | 5.7E-11 |
| rs114976176 | C | A | 0.35 | 2 | 264621 | Intron:SH3YL1 | -0.016 | 0.003 | 6.0E-09 |
| rs62106258 | C | T | 0.05 | 2 | 417167 | Intergenic | -0.045 | 0.006 | 3.3E-14 |
| rs6731872 | G | T | 0.83 | 2 | 624205 | Intergenic | 0.032 | 0.003 | 5.4E-21 |
| rs1022376 | C | T | 0.52 | 2 | 22067213 | Intergenic | -0.015 | 0.003 | 1.7E-08 |
| rs61533748 | C | T | 0.38 | 2 | 22582968 | Intergenic | 0.017 | 0.003 | 2.8E-11 |
| rs72790288 | A | G | 0.03 | 2 | 29513404 | Intron:ALK | -0.046 | 0.008 | 3.3E-09 |
| rs2710634 | C | T | 0.52 | 2 | 32808804 | Intron:BIRC6 | -0.018 | 0.003 | 3.4E-12 |
| rs62137126 | G | A | 0.12 | 2 | 44250149 | Intergenic | -0.024 | 0.004 | 1.3E-09 |
| rs1004787 | A | G | 0.55 | 2 | 45159091 | Intron:LINC01833 | 0.028 | 0.003 | 1.1E-28 |
| rs7598402 | G | C | 0.49 | 2 | 50735943 | Intron:NRXN1 | -0.015 | 0.003 | 7.4E-09 |
| rs10490159 | T | C | 0.39 | 2 | 51341259 | Intron:LOC730100 | 0.017 | 0.003 | 3.9E-11 |
| rs1518393 | C | A | 0.62 | 2 | 58171220 | Intron:VRK2 | 0.017 | 0.003 | 1.3E-10 |
| rs17616642 | G | A | 0.25 | 2 | 59022210 | Intron:LINC01122 | -0.017 | 0.003 | 2.1E-08 |
| rs6730325 | A | G | 0.61 | 2 | 59315828 | Intergenic | -0.015 | 0.003 | 2.1E-08 |
| rs2539706 | A | G | 0.53 | 2 | 59819545 | Intergenic | 0.016 | 0.003 | 2.0E-10 |
| rs7585579 | G | C | 0.50 | 2 | 60024857 | Intergenic | 0.020 | 0.003 | 5.5E-15 |
| rs1863161 | A | G | 0.56 | 2 | 60139524 | Intergenic | 0.015 | 0.003 | 2.3E-09 |
| rs359247 | T | A | 0.64 | 2 | 60477052 | Intergenic | 0.022 | 0.003 | 9.9E-17 |
| rs62180324 | A | G | 0.21 | 2 | 63416606 | Intron:WDPCP | -0.020 | 0.003 | 3.9E-10 |
| rs6750107 | A | G | 0.39 | 2 | 80748807 | Intron:CTNNA2 | 0.015 | 0.003 | 2.6E-08 |
| rs12714017 | C | T | 0.51 | 2 | 80999398 | Intergenic | 0.015 | 0.003 | 3.7E-09 |
| rs56208390 | G | A | 0.12 | 2 | 83247997 | Intergenic | 0.022 | 0.004 | 2.7E-08 |
| rs11692435 | A | G | 0.08 | 2 | 98275354 | Nonsynonymous:ACTR1B | 0.025 | 0.005 | 4.5E-08 |
| rs13392222 | C | A | 0.14 | 2 | 100672408 | Intron:AFF3 | -0.023 | 0.004 | 1.9E-10 |
| rs1901477 | G | A | 0.51 | 2 | 104126983 | Intergenic | 0.030 | 0.003 | 2.1E-31 |
| rs11889814 | C | A | 0.13 | 2 | 104432494 | Intergenic | -0.021 | 0.004 | 3.4E-08 |
| rs3811038 | C | T | 0.28 | 2 | 113240183 | Intron:TTL | 0.019 | 0.003 | 1.6E-11 |
| rs75210106 | T | C | 0.18 | 2 | 113246436 | Intron:TTL | -0.019 | 0.003 | 2.3E-08 |
| rs34399632 | G | A | 0.23 | 2 | 137571174 | Intron:THSD7B | 0.019 | 0.003 | 1.5E-10 |
| rs74697736 | A | G | 0.29 | 2 | 145412271 | Intergenic | 0.022 | 0.003 | 2.4E-15 |
| rs6756212 | T | C | 0.54 | 2 | 146140132 | Intergenic | -0.034 | 0.003 | 3.5E-40 |
| rs3076896‡ | A | G | 0.39 | 2 | 146283610 | Intergenic | 0.023 | 0.003 | 2.0E-16 |
| rs16826827 | C | T | 0.12 | 2 | 147825689 | Intergenic | -0.022 | 0.004 | 9.2E-09 |
| rs1445649 | C | T | 0.54 | 2 | 155682556 | Intron:KCNJ3 | 0.021 | 0.003 | 8.5E-16 |
| rs1722666 | T | C | 0.73 | 2 | 161816880 | Intergenic | 0.016 | 0.003 | 2.2E-08 |
| rs11678980 | A | G | 0.45 | 2 | 162101261 | Exon:LINC01806 | 0.018 | 0.003 | 5.2E-12 |
| rs12474587 | T | G | 0.43 | 2 | 162802993 | Intron:SLC4A10 | 0.024 | 0.003 | 4.8E-21 |
| rs357304 | C | T | 0.73 | 2 | 164862639 | Intergenic | 0.017 | 0.003 | 5.4E-09 |
| rs13007361 | A | G | 0.21 | 2 | 166250244 | Intergenic | 0.018 | 0.003 | 2.3E-08 |
| rs7600835 | A | G | 0.34 | 2 | 172521827 | Intergenic | -0.015 | 0.003 | 1.8E-08 |
| rs6750529 | T | C | 0.74 | 2 | 182027603 | Intron:LINC01934 | 0.020 | 0.003 | 9.3E-12 |
| rs17229285 | T | C | 0.51 | 2 | 199523122 | Intergenic | -0.015 | 0.003 | 1.3E-09 |
| rs3115418 | C | T | 0.45 | 2 | 200936399 | Intergenic | -0.014 | 0.003 | 2.8E-08 |
| rs62193862 | A | G | 0.10 | 2 | 202843875 | Intergenic | 0.024 | 0.004 | 2.0E-08 |
| rs4674916 | A | C | 0.33 | 2 | 225365635 | Intron:CUL3 | -0.018 | 0.003 | 3.1E-11 |
| rs4674993 | G | A | 0.20 | 2 | 226332033 | Intron:NYAP2 | -0.024 | 0.003 | 4.9E-14 |
| rs11713899 | C | A | 0.17 | 3 | 2365026 | Intron:CNTN4 | 0.019 | 0.003 | 3.2E-08 |
| rs748832 | G | A | 0.37 | 3 | 16851202 | Intergenic | 0.017 | 0.003 | 6.6E-11 |
| rs10446419 | G | A | 0.21 | 3 | 25725501 | Intergenic | -0.020 | 0.003 | 5.1E-10 |
| rs13319205 | A | T | 0.29 | 3 | 47800216 | Intron:SMARCC1 | 0.017 | 0.003 | 3.8E-09 |
| rs3172494 | T | G | 0.12 | 3 | 48731487 | Utr3:IP6K2 | -0.029 | 0.004 | 3.4E-13 |
| rs2526390 | T | C | 0.33 | 3 | 50192760 | Intron:SEMA3F\|SEMA3F-AS1 | 0.020 | 0.003 | 3.6E-14 |
| rs2276825 | C | T | 0.25 | 3 | 52886605 | Intron:STIMATE\|TMEM110-MUSTN1 | 0.019 | 0.003 | 1.9E-10 |
| rs2306866 | T | A | 0.61 | 3 | 53766212 | Intron:CACNA1D | -0.017 | 0.003 | 1.9E-10 |
| rs73831818 | G | A | 0.06 | 3 | 55988394 | Intron:ERC2 | 0.032 | 0.005 | 5.5E-09 |
| rs1910236 | A | G | 0.47 | 3 | 59434420 | Intergenic | 0.015 | 0.003 | 9.9E-09 |
| rs7640107 | T | C | 0.43 | 3 | 59966156 | Intron:FHIT | -0.014 | 0.003 | 3.5E-08 |
| rs2734390 | G | A | 0.37 | 3 | 60459291 | Intron:FHIT | 0.015 | 0.003 | 2.1E-08 |
| rs221988 | C | A | 0.38 | 3 | 64234307 | Intergenic | -0.015 | 0.003 | 1.4E-08 |
| rs2196356 | C | G | 0.29 | 3 | 70890288 | Intergenic | -0.019 | 0.003 | 2.5E-11 |
| rs11128203 | A | T | 0.53 | 3 | 71064431 | Intron:FOXP1 | 0.020 | 0.003 | 1.3E-15 |
| rs62246017 | A | G | 0.32 | 3 | 71483084 | Intron:FOXP1 | -0.016 | 0.003 | 3.0E-09 |
| rs4543050 | T | A | 0.82 | 3 | 74954560 | Intergenic | 0.022 | 0.003 | 1.5E-11 |
| rs6782116 | T | C | 0.42 | 3 | 77176032 | Intron:ROBO2 | -0.015 | 0.003 | 1.5E-08 |
| rs13066050 | T | C | 0.21 | 3 | 81325861 | Intergenic | 0.019 | 0.003 | 1.9E-09 |
| rs12633090 | C | G | 0.18 | 3 | 83241365 | Intergenic | -0.023 | 0.003 | 3.2E-12 |
| rs1549979 | T | C | 0.62 | 3 | 85460131 | Intron:CADM2 | -0.025 | 0.003 | 8.8E-21 |
| rs74664784 (rs35894540)† | C | T | 0.38 | 3 | 85475292 | Intron:CADM2 | -0.020 | 0.003 | 9.3E-13 |
| rs57153235 | G | T | 0.32 | 3 | 85902536 | Intron:CADM2 | -0.019 | 0.003 | 1.6E-12 |
| rs6437769 | T | C | 0.58 | 3 | 107997514 | Intergenic | 0.014 | 0.003 | 3.7E-08 |
| rs9288999 | A | G | 0.74 | 3 | 114147927 | Intron:ZBTB20 | 0.017 | 0.003 | 1.5E-09 |
| rs6438436 | T | C | 0.82 | 3 | 117822149 | Intergenic | 0.025 | 0.003 | 5.3E-14 |
| rs12053870 | G | T | 0.54 | 3 | 118302515 | Intron:LOC105374060 | 0.016 | 0.003 | 1.0E-09 |
| rs9826984 | A | G | 0.54 | 3 | 131945722 | Intergenic | -0.014 | 0.003 | 3.9E-08 |
| rs2279829 | T | C | 0.22 | 3 | 147106319 | Utr3:ZIC4 | -0.017 | 0.003 | 2.1E-08 |
| rs2319545 | A | C | 0.15 | 3 | 147719648 | Intergenic | 0.023 | 0.004 | 8.3E-11 |
| rs10935779 | T | C | 0.42 | 3 | 149543102 | Intron:RNF13 | -0.014 | 0.003 | 3.0E-08 |
| rs963354 | A | C | 0.69 | 3 | 157393770 | Intergenic | 0.015 | 0.003 | 4.2E-08 |
| rs1714521 | C | A | 0.41 | 3 | 158284861 | Intron:LOC100996447 | -0.016 | 0.003 | 3.1E-10 |
| rs1449012 | T | C | 0.46 | 3 | 159048333 | Intron:IQCJ-SCHIP1\|SCHIP1 | -0.015 | 0.003 | 1.8E-09 |
| rs9850597 | A | G | 0.82 | 3 | 161761866 | Intergenic | -0.019 | 0.003 | 1.7E-08 |
| rs1187820 | T | C | 0.44 | 3 | 173072584 | Intergenic | -0.014 | 0.003 | 2.7E-08 |
| rs16828799 | T | G | 0.16 | 3 | 173353739 | Intron:NLGN1 | 0.020 | 0.004 | 1.8E-08 |
| rs9841807 | T | C | 0.27 | 3 | 175718927 | Intergenic | 0.016 | 0.003 | 1.4E-08 |
| rs7631379 | C | T | 0.21 | 3 | 181409057 | Intron:SOX2-OT | 0.021 | 0.003 | 3.9E-11 |
| rs4140932 | A | T | 0.43 | 4 | 15458598 | Intergenic | -0.014 | 0.003 | 4.9E-08 |
| rs12642744 | T | G | 0.74 | 4 | 28027176 | Intergenic | -0.017 | 0.003 | 2.8E-08 |
| rs59537158 | T | C | 0.21 | 4 | 28246049 | Intergenic | 0.022 | 0.003 | 4.6E-13 |
| rs1389171 | A | T | 0.24 | 4 | 28822284 | Intergenic | -0.017 | 0.003 | 4.5E-09 |
| rs55944129 | C | T | 0.27 | 4 | 29082156 | Intergenic | -0.018 | 0.003 | 1.1E-09 |
| rs58400863 | A | G | 0.35 | 4 | 31184484 | Intron:LINC02497 | -0.020 | 0.003 | 4.9E-14 |
| rs7657022 | G | A | 0.49 | 4 | 35501032 | Intergenic | 0.018 | 0.003 | 7.3E-13 |
| rs55900829 (rs1399120)† | T | A | 0.33 | 4 | 35514712 | Intergenic | 0.019 | 0.003 | 5.6E-12 |
| rs112725451 | T | C | 0.17 | 4 | 68017710 | Intergenic | 0.026 | 0.003 | 1.7E-14 |
| rs1160685 | G | C | 0.45 | 4 | 94052854 | Intron:GRID2 | 0.015 | 0.003 | 2.3E-09 |
| rs1435479 | T | G | 0.29 | 4 | 94550450 | Intron:GRID2 | 0.016 | 0.003 | 5.7E-09 |
| rs3934797 | A | G | 0.18 | 4 | 112467612 | Intergenic | -0.021 | 0.003 | 1.1E-10 |
| rs71602617 | T | C | 0.22 | 4 | 136406155 | Intergenic | -0.018 | 0.003 | 2.1E-08 |
| rs7696257 | A | G | 0.37 | 4 | 137474783 | Intergenic | 0.015 | 0.003 | 6.8E-09 |
| rs13109980 | A | G | 0.33 | 4 | 140886963 | Intron:MAML3 | -0.022 | 0.003 | 3.4E-16 |
| rs1116690 | G | A | 0.74 | 4 | 143510148 | Intron:INPP4B | 0.016 | 0.003 | 2.2E-08 |
| rs13110073 | C | T | 0.40 | 4 | 147797913 | Intron:TTC29 | -0.025 | 0.003 | 3.2E-21 |
| rs28717373 | T | C | 0.36 | 4 | 147985231 | Intergenic | -0.016 | 0.003 | 6.2E-10 |
| rs62340589 | C | G | 0.20 | 4 | 176875795 | Intron:GPM6A | 0.017 | 0.003 | 4.3E-08 |
| rs12517438 | G | T | 0.54 | 5 | 30842054 | Intergenic | 0.015 | 0.003 | 1.9E-09 |
| rs35375873 | C | G | 0.11 | 5 | 43190647 | Intergenic | -0.027 | 0.004 | 3.3E-11 |
| rs986714 | T | A | 0.45 | 5 | 50821338 | Intergenic | -0.016 | 0.003 | 4.1E-10 |
| rs71592686 | C | T | 0.27 | 5 | 60121271 | Intron:ELOVL7 | 0.021 | 0.003 | 3.9E-13 |
| rs2028269 | A | G | 0.40 | 5 | 79308315 | Intron:THBS4 | 0.016 | 0.003 | 5.2E-10 |
| rs6874731 | G | T | 0.48 | 5 | 80263865 | Intron:RASGRF2 | 0.015 | 0.003 | 1.8E-09 |
| rs6452785 | T | C | 0.47 | 5 | 87685500 | Intron:TMEM161B-AS1 | -0.027 | 0.003 | 4.7E-26 |
| rs10805858 | T | A | 0.34 | 5 | 88873832 | Intergenic | 0.018 | 0.003 | 1.9E-11 |
| rs181508347 | G | T | 0.01 | 5 | 91366274 | Intergenic | 0.081 | 0.013 | 5.0E-10 |
| rs42417 | T | C | 0.69 | 5 | 94198290 | Intron:MCTP1 | 0.017 | 0.003 | 8.3E-10 |
| rs72780746 | C | T | 0.17 | 5 | 103929588 | Intergenic | -0.026 | 0.003 | 2.1E-14 |
| rs10060196 | A | C | 0.58 | 5 | 106455988 | Intergenic | 0.018 | 0.003 | 1.3E-12 |
| rs72789626 | A | T | 0.14 | 5 | 106825618 | Intron:EFNA5 | -0.026 | 0.004 | 5.1E-12 |
| rs17165769 | G | A | 0.39 | 5 | 107365642 | Intron:FBXL17 | 0.016 | 0.003 | 9.6E-10 |
| rs329124 | G | A | 0.43 | 5 | 133865452 | Intron:JADE2 | -0.016 | 0.003 | 2.0E-10 |
| rs1385108 | T | C | 0.24 | 5 | 154839646 | Intergenic | 0.019 | 0.003 | 3.8E-10 |
| rs1173461 | T | C | 0.33 | 5 | 157707571 | Intergenic | 0.017 | 0.003 | 9.5E-10 |
| rs11956866 | G | T | 0.57 | 5 | 161018271 | Intergenic | -0.015 | 0.003 | 7.8E-09 |
| rs3909281 | G | T | 0.54 | 5 | 165096435 | Intergenic | 0.021 | 0.003 | 1.6E-16 |
| rs3843905 | T | C | 0.40 | 5 | 165427280 | Intergenic | -0.015 | 0.003 | 5.4E-09 |
| rs79476395 | G | A | 0.07 | 5 | 166063680 | Intergenic | 0.033 | 0.005 | 1.0E-11 |
| rs6890961 | T | C | 0.62 | 5 | 166778503 | Intron:TENM2 | -0.019 | 0.003 | 2.1E-13 |
| rs4044321 | G | A | 0.64 | 5 | 166989513 | Intron:TENM2 | -0.023 | 0.003 | 1.8E-17 |
| rs2173019 | A | T | 0.18 | 5 | 167614971 | Intron:TENM2 | 0.028 | 0.003 | 3.0E-17 |
| rs10042827 | C | T | 0.68 | 5 | 170299916 | Intron:RANBP17 | 0.017 | 0.003 | 9.4E-10 |
| rs359431 | T | C | 0.56 | 5 | 173288534 | Intergenic | -0.014 | 0.003 | 3.2E-08 |
| rs1059490 | C | T | 0.37 | 6 | 26171250 | Utr3:HIST1H2BD | -0.019 | 0.003 | 2.2E-12 |
| rs6932350 | A | T | 0.45 | 6 | 26571629 | Intron:LOC105374988 | 0.015 | 0.003 | 5.1E-09 |
| rs1150668 | G | T | 0.42 | 6 | 28129789 | Intron:ZNF192P1 | -0.019 | 0.003 | 8.5E-13 |
| rs1632941 | C | T | 0.46 | 6 | 29796685 | Intron:HLA-G | -0.016 | 0.003 | 6.7E-10 |
| rs3218116 | T | C | 0.26 | 6 | 41901763 | Intergenic | -0.020 | 0.003 | 1.1E-11 |
| rs160631 | G | T | 0.73 | 6 | 52895230 | Intron:ICK | -0.017 | 0.003 | 1.9E-09 |
| rs7743165 | G | T | 0.50 | 6 | 67521222 | Intergenic | 0.019 | 0.003 | 4.2E-14 |
| rs79180767 (rs11753204)† | T | C | 0.25 | 6 | 67540984 | Intergenic | 0.020 | 0.003 | 7.0E-12 |
| rs10945141 | A | G | 0.26 | 6 | 69470709 | Intron:ADGRB3 | 0.018 | 0.003 | 3.6E-10 |
| rs17554906 | C | G | 0.44 | 6 | 92226609 | Intergenic | 0.014 | 0.003 | 3.1E-08 |
| rs619087 | G | A | 0.42 | 6 | 94175279 | Intergenic | 0.014 | 0.003 | 3.1E-08 |
| rs6568832 | A | G | 0.75 | 6 | 97702876 | Intron:MIR548H3\|MMS22L | 0.019 | 0.003 | 1.7E-10 |
| rs12195240 | A | G | 0.29 | 6 | 98636905 | Intergenic | 0.025 | 0.003 | 1.1E-18 |
| rs6936160 | T | C | 0.70 | 6 | 100347745 | Intergenic | 0.020 | 0.003 | 4.2E-13 |
| rs12530388 | C | A | 0.51 | 6 | 101329173 | Utr5:ASCC3 | -0.018 | 0.003 | 5.8E-13 |
| rs3800227 | G | A | 0.74 | 6 | 108994161 | Intron:FOXO3 | 0.017 | 0.003 | 3.6E-09 |
| rs118202 | T | G | 0.81 | 6 | 111658371 | Intron:REV3L | -0.037 | 0.003 | 1.9E-29 |
| rs73008357 | C | A | 0.12 | 6 | 156431856 | Intergenic | -0.022 | 0.004 | 2.4E-08 |
| rs9331343 | C | T | 0.57 | 6 | 157738258 | Intron:TMEM242 | -0.014 | 0.003 | 3.9E-08 |
| rs10698713 | A | G | 0.05 | 6 | 158882320 | Intron:TULP4 | -0.034 | 0.006 | 2.4E-09 |
| rs1737329 | G | C | 0.74 | 6 | 163807748 | Intergenic | 0.017 | 0.003 | 5.1E-09 |
| rs10272990 | C | T | 0.33 | 7 | 1703675 | Intergenic | -0.021 | 0.003 | 1.3E-14 |
| rs6948707 | G | T | 0.42 | 7 | 1870794 | Intron:MAD1L1 | 0.024 | 0.003 | 4.2E-21 |
| rs10259715 (rs4604332)†§ | A | T | 0.21 | 7 | 3329967 | Intergenic | -0.019 | 0.003 | 6.4E-09 |
| rs13237637 | C | G | 0.49 | 7 | 3503207 | Intron:SDK1 | -0.024 | 0.003 | 1.5E-20 |
| rs79631993 | C | A | 0.22 | 7 | 69432311 | Intron:AUTS2 | -0.017 | 0.003 | 3.7E-08 |
| rs7809303 | A | G | 0.33 | 7 | 69484366 | Intron:AUTS2 | -0.021 | 0.003 | 3.5E-15 |
| rs7802996 | T | C | 0.17 | 7 | 77771983 | Intron:MAGI2 | -0.021 | 0.003 | 1.1E-09 |
| rs1030015 | T | G | 0.52 | 7 | 78139581 | Intron:MAGI2 | 0.014 | 0.003 | 2.2E-08 |
| rs4727189 | C | T | 0.34 | 7 | 88442568 | Intron:ZNF804B | 0.015 | 0.003 | 3.0E-08 |
| rs76841737 | G | C | 0.10 | 7 | 91281409 | Intergenic | -0.023 | 0.004 | 3.3E-08 |
| rs11768481 | A | C | 0.34 | 7 | 96629103 | Intron:DLX6-AS1 | -0.019 | 0.003 | 5.2E-12 |
| rs1799068 | T | G | 0.38 | 7 | 97707069 | Intergenic | 0.017 | 0.003 | 2.6E-10 |
| rs13437771 | G | A | 0.16 | 7 | 99071478 | Intron:ZNF789 | -0.027 | 0.004 | 1.4E-14 |
| rs11766326 | C | T | 0.51 | 7 | 111100585 | Intron:IMMP2L | -0.018 | 0.003 | 1.8E-11 |
| rs6968380 | A | G | 0.68 | 7 | 114940159 | Intergenic | -0.023 | 0.003 | 1.1E-17 |
| rs112913817 | G | A | 0.01 | 7 | 115077394 | Intergenic | 0.078 | 0.012 | 9.3E-11 |
| rs10233018 | G | A | 0.52 | 7 | 117523709 | Intergenic | 0.025 | 0.003 | 4.8E-22 |
| rs10953957 | A | G | 0.39 | 7 | 121954709 | Intergenic | 0.014 | 0.003 | 3.7E-08 |
| rs77283305 | A | G | 0.31 | 7 | 132593831 | Intron:CHCHD3 | -0.015 | 0.003 | 3.9E-08 |
| rs10279261 | A | G | 0.62 | 7 | 133589846 | Intron:EXOC4 | -0.019 | 0.003 | 6.1E-13 |
| rs1561112 | C | T | 0.41 | 7 | 133840652 | Intron:LRGUK | -0.015 | 0.003 | 3.8E-09 |
| rs2952251 | G | A | 0.74 | 8 | 10143164 | Intron:MSRA | 0.016 | 0.003 | 4.2E-08 |
| rs4326350 | G | C | 0.49 | 8 | 10763655 | Intron:XKR6 | -0.018 | 0.003 | 5.2E-12 |
| rs11780471 | A | G | 0.06 | 8 | 27344719 | Intergenic | -0.039 | 0.005 | 1.6E-13 |
| rs11783093 | T | C | 0.16 | 8 | 27425349 | Intergenic | -0.047 | 0.003 | 2.1E-41 |
| rs1565735 | A | T | 0.20 | 8 | 27426077 | Intergenic | -0.019 | 0.003 | 1.3E-09 |
| rs7836565 | T | C | 0.72 | 8 | 52569449 | Intron:PXDNL | -0.016 | 0.003 | 4.4E-08 |
| rs13261666 | T | G | 0.52 | 8 | 59814666 | Intron:TOX | -0.020 | 0.003 | 4.4E-15 |
| rs3850736 | G | C | 0.47 | 8 | 64912021 | Intron:LOC102724623 | 0.019 | 0.003 | 6.4E-14 |
| rs2063976 | T | C | 0.66 | 8 | 91096366 | Intergenic | -0.020 | 0.003 | 7.5E-14 |
| rs6993429 | A | C | 0.45 | 8 | 92733282 | Intergenic | -0.019 | 0.003 | 9.9E-14 |
| rs6986430 | C | T | 0.22 | 8 | 93048104 | Intron:RUNX1T1 | -0.024 | 0.003 | 2.0E-15 |
| rs9987376 | G | T | 0.57 | 8 | 93190014 | Intergenic | -0.020 | 0.003 | 2.0E-15 |
| rs290601 | T | C | 0.27 | 8 | 115374642 | Intergenic | 0.016 | 0.003 | 1.1E-08 |
| rs3847244 | T | C | 0.47 | 9 | 3025368 | Intergenic | 0.019 | 0.003 | 2.6E-13 |
| rs11791671 | T | C | 0.07 | 9 | 3398679 | Intron:RFX3 | 0.028 | 0.005 | 4.2E-08 |
| rs7024924 | C | T | 0.17 | 9 | 8282399 | Intergenic | 0.019 | 0.003 | 1.9E-08 |
| rs6474609 | A | T | 0.59 | 9 | 10981069 | Intergenic | -0.016 | 0.003 | 1.7E-09 |
| rs1931431 | C | G | 0.48 | 9 | 11161799 | Intergenic | 0.018 | 0.003 | 8.6E-13 |
| rs7867822 | G | A | 0.67 | 9 | 20676454 | Intron:FOCAD | -0.015 | 0.003 | 2.8E-08 |
| rs10966092 | C | T | 0.27 | 9 | 23831658 | Intron:ELAVL2 | -0.020 | 0.003 | 1.1E-12 |
| rs10969352 | A | T | 0.50 | 9 | 29747488 | Intergenic | 0.014 | 0.003 | 1.8E-08 |
| rs4877285 | A | G | 0.67 | 9 | 81354129 | Intergenic | -0.018 | 0.003 | 2.1E-11 |
| rs1930371 | T | C | 0.24 | 9 | 81444104 | Intergenic | -0.017 | 0.003 | 7.1E-09 |
| rs2378662 | A | G | 0.54 | 9 | 86707289 | Intron:LOC101927575 | 0.015 | 0.003 | 2.7E-09 |
| rs1927901 | C | T | 0.55 | 9 | 120519111 | Intergenic | -0.014 | 0.003 | 3.1E-08 |
| rs4837631 | T | C | 0.45 | 9 | 122061948 | Intron:BRINP1 | -0.015 | 0.003 | 2.0E-09 |
| rs1759433 | A | G | 0.48 | 9 | 128073097 | Intron:GAPVD1 | 0.015 | 0.003 | 1.7E-09 |
| rs34553878 | G | A | 0.11 | 9 | 134334588 | Nonsynonymous:PRRC2B | 0.025 | 0.004 | 1.2E-09 |
| rs7026534 | G | T | 0.70 | 9 | 134907263 | Intron:MED27 | -0.017 | 0.003 | 2.7E-09 |
| rs10858334 | G | C | 0.14 | 9 | 137989785 | Utr3:OLFM1 | 0.023 | 0.004 | 1.2E-09 |
| rs10905461 | C | T | 0.75 | 10 | 8803551 | Intergenic | -0.016 | 0.003 | 2.4E-08 |
| rs7920501 | A | T | 0.47 | 10 | 10043159 | Intergenic | -0.016 | 0.003 | 1.3E-09 |
| rs1291821 | G | A | 0.53 | 10 | 11133823 | Intron:CELF2\|CELF2-AS2 | 0.014 | 0.003 | 1.4E-08 |
| rs11258417 | T | C | 0.39 | 10 | 13533053 | Intron:BEND7 | -0.015 | 0.003 | 2.7E-08 |
| rs7072776 | G | A | 0.71 | 10 | 22032942 | Intergenic | -0.022 | 0.003 | 5.7E-15 |
| rs2796793 | A | G | 0.45 | 10 | 36634124 | Intergenic | 0.014 | 0.003 | 1.6E-08 |
| rs1733760 | C | T | 0.51 | 10 | 56698174 | Intron:PCDH15 | 0.015 | 0.003 | 6.7E-09 |
| rs7921378 | C | G | 0.48 | 10 | 63674885 | Intron:ARID5B | -0.023 | 0.003 | 6.1E-20 |
| rs7901883 | A | G | 0.23 | 10 | 103186838 | Intron:BTRC | -0.019 | 0.003 | 2.0E-10 |
| rs11594623 | C | T | 0.23 | 10 | 103960351 | Intergenic | 0.027 | 0.003 | 7.5E-20 |
| rs11191269 | G | C | 0.19 | 10 | 104120522 | Intron:GBF1 | 0.018 | 0.003 | 4.6E-08 |
| rs28408682 | G | A | 0.60 | 10 | 104403310 | Intergenic | 0.017 | 0.003 | 1.4E-10 |
| rs12244388 | A | G | 0.35 | 10 | 104640052 | Intron:AS3MT\|BORCS7-ASMT | 0.026 | 0.003 | 4.3E-22 |
| rs111842178 (rs3740387)†§ | G | A | 0.23 | 10 | 104852121 | Intron:NT5C2 | 0.022 | 0.003 | 2.2E-12 |
| rs34970111 | T | C | 0.46 | 10 | 106078937 | Intron:ITPRIP | -0.015 | 0.003 | 1.3E-08 |
| rs9787523 | C | T | 0.42 | 10 | 106460460 | Intron:SORCS3 | -0.016 | 0.003 | 1.4E-09 |
| rs11192347 | A | G | 0.10 | 10 | 106929313 | Intron:SORCS3 | -0.026 | 0.004 | 6.2E-10 |
| rs10885480 | C | T | 0.28 | 10 | 115378364 | Intron:NRAP | -0.019 | 0.003 | 3.8E-11 |
| rs4752018 | A | C | 0.23 | 10 | 118678712 | Intron:SHTN1 | 0.019 | 0.003 | 4.4E-10 |
| rs9423279 | G | C | 0.65 | 10 | 125680419 | Intergenic | -0.019 | 0.003 | 3.1E-12 |
| rs6265 | T | C | 0.19 | 11 | 27679916 | Nonsynonymous:BDNF | -0.029 | 0.003 | 2.8E-19 |
| rs4275621 | G | A | 0.38 | 11 | 28652996 | Intergenic | -0.021 | 0.003 | 3.8E-16 |
| rs62618693 | T | C | 0.04 | 11 | 32956492 | Nonsynonymous:QSER1 | -0.035 | 0.006 | 2.1E-08 |
| rs2939756 | A | G | 0.48 | 11 | 41436297 | Intron:LRRC4C | -0.016 | 0.003 | 7.5E-10 |
| rs1381775 | C | T | 0.71 | 11 | 42442826 | Intergenic | -0.016 | 0.003 | 2.8E-08 |
| rs2959084 | A | G | 0.70 | 11 | 46078656 | Intron:PHF21A | 0.017 | 0.003 | 9.8E-10 |
| rs3740977 | C | T | 0.17 | 11 | 46393574 | Intron:DGKZ | 0.019 | 0.003 | 1.2E-08 |
| rs61886926 | T | C | 0.38 | 11 | 64133552 | Intron:RPS6KA4 | -0.018 | 0.003 | 7.3E-12 |
| rs61884449 | T | C | 0.15 | 11 | 64485193 | Intron:NRXN2 | 0.020 | 0.004 | 2.3E-08 |
| rs644740 | T | C | 0.46 | 11 | 65561468 | Intron:OVOL1 | -0.014 | 0.003 | 3.7E-08 |
| rs7943721 | A | G | 0.83 | 11 | 73309393 | Intergenic | -0.021 | 0.003 | 3.6E-10 |
| rs7929518 | G | A | 0.77 | 11 | 85980958 | Intron:EED | 0.019 | 0.003 | 2.6E-10 |
| rs586699 | A | G | 0.54 | 11 | 92289734 | Intron:FAT3 | -0.015 | 0.003 | 7.3E-09 |
| rs76460663 | G | C | 0.04 | 11 | 111979741 | Intergenic | -0.042 | 0.006 | 4.2E-11 |
| rs2155646 | C | T | 0.40 | 11 | 112912811 | Intron:NCAM1 | 0.038 | 0.003 | 9.4E-48 |
| rs78239456 (rs11214501)† | T | A | 0.38 | 11 | 112984491 | Intron:NCAM1 | -0.018 | 0.003 | 9.4E-12 |
| rs1713676 | G | A | 0.52 | 11 | 113660576 | Intergenic | -0.017 | 0.003 | 5.4E-11 |
| rs238896 | A | G | 0.49 | 11 | 113994505 | Intron:ZBTB16 | -0.017 | 0.003 | 3.7E-11 |
| rs540860 | G | A | 0.54 | 11 | 121530888 | Intergenic | 0.018 | 0.003 | 5.8E-12 |
| rs1944689 | T | G | 0.79 | 11 | 121634334 | Intergenic | 0.018 | 0.003 | 1.3E-08 |
| rs1834306 | G | A | 0.58 | 11 | 122023187 | Intron:MIR100HG | -0.014 | 0.003 | 2.0E-08 |
| rs1106363 | T | C | 0.34 | 11 | 131966264 | Intron:NTM | 0.017 | 0.003 | 9.2E-11 |
| rs2010921 | A | G | 0.31 | 11 | 132098205 | Intron:NTM | 0.017 | 0.003 | 2.5E-10 |
| rs11057005 | G | A | 0.44 | 12 | 16748721 | Intron:LMO3 | -0.016 | 0.003 | 9.1E-10 |
| rs13906 | T | C | 0.11 | 12 | 49952394 | Utr3:MCRS1 | -0.025 | 0.004 | 2.0E-09 |
| rs4759229 | G | A | 0.66 | 12 | 56474480 | Intron:ERBB3 | 0.016 | 0.003 | 6.5E-09 |
| rs7969559 | G | A | 0.71 | 12 | 69655167 | Intron:CPSF6 | -0.017 | 0.003 | 1.5E-09 |
| rs7134009 | C | T | 0.29 | 12 | 75263193 | Intergenic | -0.016 | 0.003 | 4.3E-08 |
| rs77215829 | C | A | 0.13 | 12 | 112618346 | Intron:HECTD4 | -0.024 | 0.004 | 2.0E-10 |
| rs1109480 | A | G | 0.38 | 12 | 121083279 | Intron:CABP1 | -0.017 | 0.003 | 1.8E-10 |
| rs11611651 | A | G | 0.09 | 12 | 133380790 | Intron:GOLGA3 | 0.027 | 0.005 | 2.1E-09 |
| rs17197663 | A | G | 0.13 | 13 | 38172867 | Utr5:POSTN | -0.022 | 0.004 | 2.1E-08 |
| rs4264267 | T | C | 0.53 | 13 | 38359676 | Intron:TRPC4 | 0.015 | 0.003 | 6.8E-09 |
| rs61959481 | A | G | 0.21 | 13 | 55834929 | Intergenic | -0.020 | 0.003 | 8.0E-11 |
| rs3098272 | C | A | 0.80 | 13 | 55931424 | Intergenic | -0.018 | 0.003 | 2.1E-08 |
| rs9538162 | C | T | 0.42 | 13 | 59265043 | Intergenic | 0.017 | 0.003 | 1.8E-11 |
| rs1413119 | T | C | 0.40 | 13 | 59339281 | Intergenic | -0.015 | 0.003 | 4.8E-09 |
| rs56367474 | T | C | 0.30 | 13 | 59454139 | Intergenic | -0.017 | 0.003 | 4.2E-10 |
| rs55786907 | G | A | 0.16 | 13 | 59871584 | Intergenic | 0.019 | 0.003 | 1.8E-08 |
| rs4886207 | C | T | 0.64 | 13 | 60705792 | Intron:DIAPH3 | -0.016 | 0.003 | 8.8E-10 |
| rs9540731 | T | C | 0.51 | 13 | 66949370 | Intron:PCDH9 | -0.018 | 0.003 | 3.4E-12 |
| rs9545155 | C | T | 0.48 | 13 | 80191873 | Intergenic | -0.016 | 0.003 | 3.0E-10 |
| rs1772572 | A | C | 0.32 | 13 | 81191176 | Intergenic | -0.017 | 0.003 | 5.6E-10 |
| rs75674569 | A | G | 0.10 | 13 | 96823724 | Intron:HS6ST3 | -0.025 | 0.004 | 2.6E-09 |
| rs7333559 | A | G | 0.78 | 13 | 100546450 | Intron:CLYBL\|LOC101927437 | -0.023 | 0.003 | 5.9E-14 |
| rs1108130 | A | T | 0.21 | 13 | 100648356 | Exon:LINC00554 | 0.024 | 0.003 | 1.6E-14 |
| rs12855717 | T | C | 0.54 | 13 | 101252635 | Intergenic | 0.016 | 0.003 | 1.2E-09 |
| rs12878369 | A | C | 0.41 | 14 | 28346502 | Intergenic | 0.017 | 0.003 | 1.6E-11 |
| rs2145451‡ | C | T | 0.19 | 14 | 29316842 | Intergenic | -0.020 | 0.003 | 5.4E-10 |
| rs9323328 | G | A | 0.54 | 14 | 58653514 | Intergenic | -0.014 | 0.003 | 2.6E-08 |
| rs1811739 | A | G | 0.25 | 14 | 77529375 | Intron:LINC02288 | 0.018 | 0.003 | 6.0E-10 |
| rs8005334 | G | T | 0.36 | 14 | 79563654 | Intron:NRXN3 | 0.017 | 0.003 | 3.4E-10 |
| rs34940743 | G | A | 0.35 | 14 | 80102233 | Intron:NRXN3 | 0.016 | 0.003 | 2.8E-09 |
| rs2925128 | T | C | 0.39 | 14 | 98362355 | Intergenic | 0.017 | 0.003 | 3.7E-10 |
| rs1381287 | T | C | 0.47 | 14 | 98597552 | Intergenic | 0.018 | 0.003 | 1.8E-12 |
| rs55913542 | T | G | 0.18 | 14 | 99693843 | Intron:BCL11B | 0.019 | 0.003 | 3.3E-08 |
| rs1435672 | C | T | 0.56 | 15 | 36399479 | Intergenic | 0.014 | 0.003 | 3.8E-08 |
| rs281296 | A | G | 0.36 | 15 | 47685010 | Intron:SEMA6D | 0.025 | 0.003 | 1.6E-20 |
| rs1435741 | A | G | 0.43 | 15 | 47935843 | Intron:SEMA6D | 0.018 | 0.003 | 1.1E-12 |
| rs56902655 | G | T | 0.14 | 15 | 63898709 | Intergenic | -0.022 | 0.004 | 4.1E-09 |
| rs2289791 | T | G | 0.25 | 15 | 67476952 | Intron:SMAD3 | -0.018 | 0.003 | 2.0E-09 |
| rs60833441 | G | A | 0.46 | 15 | 74048768 | Intergenic | -0.014 | 0.003 | 2.3E-08 |
| rs62007780 | T | G | 0.42 | 15 | 78025464 | Intron:LINGO1 | -0.016 | 0.003 | 7.5E-10 |
| rs12442563‡ | T | G | 0.22 | 15 | 83893243 | Intergenic | -0.023 | 0.003 | 3.1E-14 |
| rs4310804 | G | C | 0.25 | 15 | 96858409 | Intron:NR2F2-AS1 | -0.018 | 0.003 | 7.6E-10 |
| rs8027457 | C | T | 0.51 | 15 | 99204101 | Intron:IGF1R | 0.015 | 0.003 | 1.9E-09 |
| rs1139897 | A | G | 0.23 | 16 | 720986 | Nonsynonymous:RHOT2 | -0.024 | 0.003 | 1.8E-15 |
| rs11076962 | C | T | 0.28 | 16 | 5811367 | Intergenic | 0.018 | 0.003 | 1.2E-10 |
| rs7192140 | C | T | 0.50 | 16 | 10173748 | Intron:GRIN2A | -0.017 | 0.003 | 3.4E-11 |
| rs9922607 | T | C | 0.20 | 16 | 17570220 | Intergenic | -0.022 | 0.003 | 3.4E-12 |
| rs9941217 | G | C | 0.35 | 16 | 18050926 | Intergenic | -0.019 | 0.003 | 3.5E-12 |
| rs7188873 | G | A | 0.61 | 16 | 24727064 | Intron:TNRC6A | 0.020 | 0.003 | 8.5E-15 |
| rs6497840 | A | G | 0.71 | 16 | 25351633 | Intergenic | 0.023 | 0.003 | 2.0E-15 |
| rs4785187 | A | G | 0.22 | 16 | 49766772 | Intron:ZNF423 | 0.020 | 0.003 | 6.6E-11 |
| rs8050598 | T | C | 0.25 | 16 | 49891964 | Intergenic | 0.019 | 0.003 | 1.8E-10 |
| rs12918191 | G | A | 0.24 | 16 | 50945156 | Intergenic | -0.020 | 0.003 | 3.1E-11 |
| rs9302604 | G | A | 0.44 | 16 | 69576894 | Intergenic | 0.019 | 0.003 | 3.3E-13 |
| rs9936784 | G | T | 0.53 | 16 | 72230694 | Intergenic | 0.014 | 0.003 | 4.3E-08 |
| rs62052916 | T | A | 0.07 | 16 | 72574550 | Intron:LINC01572 | -0.032 | 0.005 | 1.6E-10 |
| rs4788676 | C | T | 0.23 | 16 | 72950468 | Intron:ZFHX3 | -0.018 | 0.003 | 4.9E-09 |
| rs61537885 | C | T | 0.04 | 16 | 75620118 | Intergenic | -0.040 | 0.007 | 8.1E-09 |
| rs117657830 | G | A | 0.04 | 16 | 75766873 | Intergenic | -0.038 | 0.006 | 3.2E-09 |
| rs1050847 | T | C | 0.56 | 16 | 87443734 | Utr3:ZCCHC14 | -0.015 | 0.003 | 7.4E-09 |
| rs11642231 | A | G | 0.37 | 16 | 89608702 | Intron:SPG7 | -0.016 | 0.003 | 3.4E-09 |
| rs4790874 | T | C | 0.53 | 17 | 1995177 | Intron:SMG6 | 0.017 | 0.003 | 8.4E-12 |
| rs11078713 | G | A | 0.42 | 17 | 7795972 | Intron:CHD3 | -0.015 | 0.003 | 1.6E-08 |
| rs28441558 | C | T | 0.06 | 17 | 7803118 | Intron:CHD3 | -0.036 | 0.006 | 1.2E-10 |
| rs11651955 | A | G | 0.50 | 17 | 16235462 | Intergenic | -0.014 | 0.003 | 3.7E-08 |
| rs67777803 | T | G | 0.17 | 17 | 27323322 | Intron:SEZ6 | -0.025 | 0.003 | 3.2E-13 |
| rs2344976 | C | T | 0.61 | 17 | 30685935 | Intron:ZNF207 | -0.015 | 0.003 | 8.0E-09 |
| rs3764351 | A | G | 0.66 | 17 | 37824339 | Intron:PNMT | -0.015 | 0.003 | 3.9E-08 |
| rs72836318‡ | C | T | 0.25 | 17 | 44121579 | Intron:KANSL1 | -0.017 | 0.003 | 7.0E-09 |
| rs17692129 | T | C | 0.33 | 17 | 44793283 | Intron:NSF | 0.020 | 0.003 | 4.6E-13 |
| rs75919030 | C | T | 0.27 | 17 | 50193197 | Intron:CA10 | -0.021 | 0.003 | 3.4E-13 |
| rs2938134 | A | C | 0.67 | 17 | 50243397 | Intergenic | -0.018 | 0.003 | 3.1E-10 |
| rs2587507 | C | T | 0.50 | 17 | 77790135 | Intergenic | -0.015 | 0.003 | 8.7E-09 |
| rs34342129 | C | T | 0.51 | 18 | 5872472 | Intergenic | -0.014 | 0.003 | 2.1E-08 |
| rs4476253 | A | G | 0.24 | 18 | 25253297 | Intergenic | -0.018 | 0.003 | 5.8E-10 |
| rs7505855 | T | C | 0.59 | 18 | 31696075 | Intron:NOL4 | -0.017 | 0.003 | 5.3E-11 |
| rs8096225 | C | A | 0.70 | 18 | 36921851 | Intron:MIR924HG | 0.016 | 0.003 | 2.6E-08 |
| rs67050670 | G | A | 0.23 | 18 | 39297254 | Intergenic | -0.020 | 0.003 | 2.3E-11 |
| rs2359180 | G | A | 0.37 | 18 | 41314171 | Intergenic | -0.014 | 0.003 | 5.0E-08 |
| rs72898831 | G | A | 0.16 | 18 | 42658643 | Intergenic | -0.024 | 0.004 | 4.1E-12 |
| rs8083764 | T | G | 0.31 | 18 | 49874515 | Intron:DCC | -0.016 | 0.003 | 8.0E-09 |
| rs1373178 | G | T | 0.59 | 18 | 49967811 | Intron:DCC | -0.020 | 0.003 | 4.2E-15 |
| rs62098013 | A | G | 0.37 | 18 | 50863861 | Intron:DCC | 0.018 | 0.003 | 2.2E-11 |
| rs72938304 | A | G | 0.11 | 18 | 53661743 | Intergenic | -0.027 | 0.004 | 1.4E-11 |
| rs11872397 | A | G | 0.25 | 18 | 72535282 | Intron:ZNF407 | -0.017 | 0.003 | 5.2E-09 |
| rs71367544 | T | C | 0.20 | 18 | 77574374 | Intergenic | 0.021 | 0.003 | 8.5E-11 |
| rs76608582 | A | C | 0.05 | 19 | 4474725 | Intron:HDGFL2 | -0.035 | 0.006 | 4.9E-09 |
| rs10853981 | A | G | 0.33 | 19 | 4965064 | Intergenic | 0.015 | 0.003 | 4.9E-08 |
| rs113230003 | A | G | 0.26 | 19 | 18460956 | Intron:PGPEP1 | -0.019 | 0.003 | 1.1E-10 |
| rs8103660 | C | T | 0.35 | 19 | 18566395 | Intron:ELL | 0.016 | 0.003 | 3.0E-09 |
| rs117734003 | C | G | 0.07 | 19 | 51129745 | Intron:SYT3 | 0.030 | 0.005 | 2.6E-09 |
| rs1126757 | T | C | 0.47 | 19 | 55879872 | Synonymous:IL11 | 0.014 | 0.003 | 2.9E-08 |
| rs6050446 | G | A | 0.97 | 20 | 25195509 | Nonsynonymous:ENTPD6 | 0.054 | 0.008 | 8.8E-13 |
| rs6058782 | T | C | 0.91 | 20 | 29946968 | Intergenic | 0.030 | 0.004 | 1.8E-11 |
| rs1555445 | T | A | 0.32 | 20 | 31175258 | Upstream:NOL4L-DT | 0.019 | 0.003 | 7.8E-12 |
| rs6073075 | A | T | 0.82 | 20 | 42015801 | Intergenic | -0.019 | 0.003 | 2.4E-08 |
| rs910912 | C | T | 0.74 | 20 | 54462393 | Intergenic | -0.017 | 0.003 | 7.8E-09 |
| rs6011779 | T | C | 0.81 | 20 | 61984317 | Intron:CHRNA4 | -0.019 | 0.003 | 2.8E-09 |
| rs3810496 | C | T | 0.62 | 20 | 62406886 | Intron:ZBTB46 | 0.016 | 0.003 | 1.5E-09 |
| rs4818005 | A | G | 0.58 | 21 | 40588819 | Intron:BRWD1 | -0.020 | 0.003 | 1.1E-14 |
| rs139896 | C | T | 0.65 | 22 | 38397797 | Intron:POLR2F | 0.015 | 0.003 | 7.1E-09 |
| rs4822102 | T | C | 0.62 | 22 | 42698430 | Intergenic | -0.017 | 0.003 | 2.8E-10 |
| rs9627272 | C | G | 0.41 | 22 | 46442288 | Intergenic | -0.015 | 0.003 | 2.4E-09 |

Chr, chromosome; EA, effect allele; EAF, effect allele frequency; NA, non-effect allele; SE, standard error; SNP, single nucleotide polymorphism.

* The beta coefficients represent the change in the probability of being an individual had ever smoked regularly for each additional effect allele.

†These SNPs were not available in the atrial fibrillation database. The proxy SNPs were founded, which shown in the parentheses.

‡These SNPs were not found in the atrial fibrillation database and no appropriate proxy SNPs were available.

§These SNPs were not available in the heavy alcohol drink database in the multivariable MR analysis.

## Supplementary Table 3. Characteristics of the genetic variants associated with age at initiation of regular smoking

| SNP | EA | NA | EAF | Chr | Position | Gene | Beta* | Se | P-value |
| --- | --- | --- | --- | --- | --- | --- | --- | --- | --- |
| rs72853300 | T | C | 0.15 | 2 | 145638766 | Intron:TEX41 | 0.019 | 0.003 | 1.8E-08 |
| rs12611472‡ | C | T | 0.30 | 2 | 225353649 | Intron:CUL3 | 0.018 | 0.003 | 3.5E-12 |
| rs7559982 | A | T | 0.56 | 2 | 63622309 | Intron:WDPCP | -0.017 | 0.002 | 1.7E-12 |
| rs11915747 | G | C | 0.35 | 3 | 85699040 | Intron:CADM2 | 0.020 | 0.003 | 1.6E-15 |
| rs13136239 | A | G | 0.34 | 4 | 140908755 | Intron:MAML3 | 0.015 | 0.003 | 6.3E-09 |
| rs2471711 | T | C | 0.15 | 4 | 28589079 | Intergenic | -0.019 | 0.003 | 1.2E-08 |
| rs624833 | G | T | 0.30 | 4 | 2881256 | Intron:ADD1 | 0.016 | 0.003 | 2.4E-09 |
| rs7682598 | G | A | 0.77 | 4 | 68000888 | Intergenic | 0.017 | 0.003 | 2.1E-09 |
| rs1403174 | T | A | 0.58 | 7 | 2032865 | Intron:MAD1L1 | 0.016 | 0.002 | 2.5E-10 |
| rs11780471 | A | G | 0.06 | 8 | 27344719 | Intergenic | 0.033 | 0.005 | 9.4E-11 |

Chr, chromosome; EA, effect allele; EAF, effect allele frequency; NA, non-effect allele; SE, standard error; SNP, single nucleotide polymorphism.

*The beta coefficients represent the change in age at initiation of regular smoking for each additional effect allele.

‡These SNPs were not found in the atrial fibrillation database and no appropriate proxy SNPs were available.

## Supplementary Table 4. Characteristics of the genetic variants associated with cigarettes per day

| **SNP** | **EA** | **NA** | **EAF** | **Chr** | **Position** | **Gene** | **Beta*** | **Se** | **P-value** |
| --- | --- | --- | --- | --- | --- | --- | --- | --- | --- |
| rs11264100 | G | A | 0.88 | 1 | 35591626 | Intergenic | -0.022 | 0.004 | 2.2E-09 |
| rs2072659 | G | C | 0.10 | 1 | 154548521 | Utr3:CHRNB2 | -0.030 | 0.004 | 2.5E-13 |
| rs34973462 | T | C | 0.33 | 1 | 175993820 | Intron:RFWD2 | 0.015 | 0.003 | 5.9E-09 |
| rs7599488 | T | C | 0.44 | 2 | 60718347 | Intron:BCL11A | 0.014 | 0.002 | 9.0E-09 |
| rs78408772 | T | C | 0.10 | 2 | 62710608 | Intergenic | -0.022 | 0.004 | 4.5E-08 |
| rs10204824 | G | A | 0.64 | 2 | 148372720 | Intergenic | -0.018 | 0.003 | 1.4E-12 |
| rs2084533 | T | C | 0.32 | 3 | 16872929 | Intergenic | 0.016 | 0.003 | 6.5E-10 |
| rs7431710 | A | G | 0.65 | 3 | 48935583 | Intron:SLC25A20 | -0.018 | 0.003 | 1.0E-12 |
| rs2236951 | C | T | 0.20 | 3 | 50421081 | Intron:CACNA2D2 | -0.017 | 0.003 | 1.6E-08 |
| rs699165 | G | A | 0.75 | 3 | 136224697 | Intron:STAG1 | 0.016 | 0.003 | 8.1E-09 |
| rs28813180 | A | G | 0.50 | 3 | 158083918 | Intron:RSRC1 | -0.015 | 0.002 | 2.0E-10 |
| rs1024323 | T | C | 0.38 | 4 | 3006043 | Nonsynonymous:GRK4 | -0.014 | 0.003 | 8.7E-09 |
| rs11940255 | A | G | 0.72 | 4 | 67086288 | Intergenic | -0.017 | 0.003 | 2.2E-10 |
| rs10454798 | T | G | 0.25 | 4 | 67980830 | Intergenic | 0.016 | 0.003 | 1.5E-08 |
| rs7766641 | A | G | 0.27 | 6 | 26184102 | Nonsynonymous:HIST1H2BE | -0.017 | 0.003 | 2.9E-10 |
| rs215600 | A | G | 0.65 | 7 | 32333642 | Intron:PDE1C | -0.024 | 0.003 | 4.0E-21 |
| rs62447179 | A | G | 0.30 | 7 | 50339609 | Intergenic | -0.015 | 0.003 | 9.7E-09 |
| rs2741351 | C | A | 0.83 | 8 | 27418040 | Intergenic | 0.018 | 0.003 | 8.8E-09 |
| rs73229090 | A | C | 0.11 | 8 | 27442127 | Intergenic | 0.026 | 0.004 | 1.1E-11 |
| rs13253502 | A | G | 0.41 | 8 | 42442018 | Intergenic | -0.014 | 0.002 | 2.3E-08 |
| rs4236926 | G | T | 0.77 | 8 | 42578059 | Intron:CHRNB3 | 0.034 | 0.003 | 7.7E-33 |
| rs790564 | C | A | 0.73 | 8 | 64604218 | Intergenic | -0.018 | 0.003 | 1.2E-10 |
| rs75596189 | T | C | 0.11 | 9 | 136468701 | Intergenic | 0.036 | 0.004 | 1.8E-20 |
| rs3025383 | C | T | 0.19 | 9 | 136502369 | Intron:DBH | -0.031 | 0.003 | 9.8E-24 |
| rs7951365 | C | T | 0.31 | 11 | 16377044 | Intron:SOX6 | 0.018 | 0.003 | 1.5E-11 |
| rs10742683 | A | G | 0.42 | 11 | 43667625 | Intergenic | -0.013 | 0.002 | 4.8E-08 |
| rs113001570 | T | A | 0.07 | 11 | 46737412 | Intergenic | 0.030 | 0.005 | 1.0E-09 |
| rs7125588 | G | A | 0.43 | 11 | 113436072 | Intergenic | -0.017 | 0.002 | 6.5E-12 |
| rs11846838 | A | G | 0.33 | 14 | 104184737 | Intron:ZFYVE21 | 0.015 | 0.003 | 5.0E-09 |
| rs1115019 | C | T | 0.79 | 15 | 57141231 | Intergenic | -0.018 | 0.003 | 2.3E-09 |
| rs632811 | G | A | 0.33 | 15 | 59155050 | Intergenic | -0.018 | 0.003 | 1.7E-10 |
| rs4886550‡ | G | A | 0.29 | 15 | 78243579 | Intergenic | -0.020 | 0.003 | 4.6E-09 |
| rs12438181 | A | G | 0.22 | 15 | 78812098 | Intron:HYKK | -0.019 | 0.003 | 5.0E-10 |
| rs10519203 | A | G | 0.66 | 15 | 78814046 | Intron:HYKK | -0.094 | 0.003 | 3.1E-286 |
| rs28438420 | T | A | 0.55 | 15 | 78836288 | Intron:PSMA4 | 0.018 | 0.002 | 1.3E-12 |
| rs72740955 | T | C | 0.34 | 15 | 78849779 | Intergenic | 0.032 | 0.003 | 2.4E-34 |
| rs146009840 | T | A | 0.34 | 15 | 78906177 | Intron:CHRNA3 | 0.022 | 0.003 | 2.0E-17 |
| rs28681284 | T | C | 0.21 | 15 | 78908565 | Intron:CHRNA3 | -0.049 | 0.003 | 2.1E-58 |
| rs8040868 | C | T | 0.40 | 15 | 78911181 | Synonymous:CHRNA3 | 0.016 | 0.003 | 1.8E-10 |
| rs3743063 | C | A | 0.56 | 15 | 79065171 | Intron:ADAMTS7 | -0.017 | 0.002 | 1.5E-11 |
| rs182317 | T | G | 0.36 | 15 | 89943601 | Intergenic | -0.016 | 0.003 | 1.3E-09 |
| rs1592485 | A | C | 0.61 | 16 | 52093549 | Intron:C16orf97 | -0.016 | 0.003 | 1.1E-10 |
| rs12924872 | T | C | 0.46 | 16 | 69552215 | Intergenic | -0.013 | 0.002 | 4.4E-08 |
| rs258321 | G | A | 0.43 | 16 | 89756473 | Intron:CDK10 | 0.016 | 0.002 | 1.5E-10 |
| rs4144686 | A | G | 0.17 | 18 | 53251725 | Intron:TCF4 | -0.019 | 0.003 | 1.4E-08 |
| rs4485470 | A | G | 0.59 | 18 | 62125063 | Intergenic | -0.015 | 0.002 | 7.1E-10 |
| rs59208569 | C | G | 0.83 | 19 | 4044424 | Utr3:ZBTB7A | 0.020 | 0.003 | 2.5E-10 |
| rs143200968 | C | G | 0.03 | 19 | 41338847 | Intergenic | -0.086 | 0.008 | 7.0E-28 |
| rs56113850 | C | T | 0.56 | 19 | 41353107 | Intron:CYP2A6 | 0.052 | 0.002 | 4.0E-99 |
| rs8192726 | A | C | 0.07 | 19 | 41354496 | Intron:CYP2A6 | -0.039 | 0.005 | 8.4E-16 |
| rs117824460 | G | A | 0.03 | 19 | 41371480 | Intergenic | -0.095 | 0.008 | 7.7E-35 |
| rs6078373 | A | G | 0.40 | 20 | 11863500 | Intergenic | 0.016 | 0.002 | 9.4E-11 |
| rs1737894 | G | C | 0.41 | 20 | 31054702 | Intron:NOL4L | 0.017 | 0.002 | 9.9E-12 |
| rs2273500 | C | T | 0.15 | 20 | 61986949 | Intron:CHRNA4 | 0.036 | 0.003 | 3.5E-26 |
| rs7281463 | C | A | 0.41 | 21 | 40520783 | Intergenic | 0.014 | 0.002 | 3.2E-08 |

Chr, chromosome; EA, effect allele; EAF, effect allele frequency; NA, non-effect allele; SE, standard error; SNP, single nucleotide polymorphism.

*The beta coefficients represent the change in cigarettes per day for each additional effect allele.

‡These SNPs were not found in the atrial fibrillation database and no appropriate proxy SNPs were available.

## Supplementary Table 5. Characteristics of the genetic variants associated with smoking cessation

| **SNP** | **EA** | **NA** | **EAF** | **Chr** | **Position** | **Gene** | **Beta*** | **Se** | **P-value** |
| --- | --- | --- | --- | --- | --- | --- | --- | --- | --- |
| rs112187834 | A | T | 0.14 | 2 | 23953454 | Intergenic | 0.033 | 0.006 | 2.8E-09 |
| rs7617480 | C | A | 0.77 | 3 | 49210732 | Intron:KLHDC8B | -0.033 | 0.005 | 1.7E-12 |
| rs12203592 | T | C | 0.18 | 6 | 396321 | Intron:IRF4 | -0.029 | 0.005 | 1.2E-08 |
| rs707968 | G | A | 0.68 | 6 | 35058117 | Utr3:ANKS1A | 0.023 | 0.004 | 2.8E-08 |
| rs7778443 | C | T | 0.62 | 7 | 32314690 | Intron:PDE1C | -0.023 | 0.004 | 1.0E-08 |
| rs1565735 | A | T | 0.20 | 8 | 27426077 | Intergenic | -0.035 | 0.005 | 1.5E-12 |
| rs60749569 | T | A | 0.08 | 8 | 42602668 | Intergenic | -0.040 | 0.007 | 2.7E-08 |
| rs12378015 | A | G | 0.30 | 9 | 127917257 | Intron:PPP6C | -0.028 | 0.004 | 8.3E-11 |
| rs9409844 | A | G | 0.05 | 9 | 136461851 | Intergenic | -0.059 | 0.009 | 4.4E-10 |
| rs3025327 | C | G | 0.11 | 9 | 136467344 | Intergenic | 0.079 | 0.006 | 1.2E-35 |
| rs10821523 | C | A | 0.54 | 9 | 136473572 | Intergenic | 0.026 | 0.004 | 2.3E-11 |
| rs1611124 | T | G | 0.07 | 9 | 136509275 | Intron:DBH | -0.045 | 0.008 | 5.3E-09 |
| rs7109376 | A | T | 0.28 | 11 | 16372431 | Intron:SOX6 | 0.028 | 0.004 | 1.1E-10 |
| rs591143 | T | C | 0.59 | 15 | 47647755 | Intron:SEMA6D | -0.024 | 0.004 | 1.1E-09 |
| rs3866543 | G | T | 0.52 | 15 | 76629609 | Intron:ISL2 | 0.022 | 0.004 | 1.4E-08 |
| rs518425 | G | A | 0.29 | 15 | 78883813 | Intron:CHRNA5 | -0.031 | 0.004 | 1.7E-12 |
| rs145580088 | G | A | 0.02 | 19 | 41342842 | Intergenic | 0.091 | 0.013 | 9.5E-13 |
| rs56113850 | C | T | 0.57 | 19 | 41353107 | Intron:CYP2A6 | -0.058 | 0.004 | 1.6E-48 |
| rs117824460 | G | A | 0.03 | 19 | 41371480 | Intergenic | 0.086 | 0.012 | 1.1E-12 |
| rs59586387 | G | C | 0.07 | 19 | 41375030 | Intergenic | 0.051 | 0.008 | 3.4E-11 |
| rs6011779 | T | C | 0.81 | 20 | 61984317 | Intron:CHRNA4 | -0.050 | 0.005 | 9.9E-24 |
| rs4809543 | A | G | 0.08 | 20 | 61986950 | Intron:CHRNA4 | 0.044 | 0.007 | 2.4E-09 |
| rs6089904 | T | A | 0.05 | 20 | 62018289 | Intergenic | -0.064 | 0.009 | 4.0E-12 |
| rs9607805 | T | C | 0.73 | 22 | 41854446 | Intergenic | 0.030 | 0.004 | 1.4E-11 |

Chr, chromosome; EA, effect allele; EAF, effect allele frequency; NA, non-effect allele; SE, standard error; SNP, single nucleotide polymorphism.

*The beta coefficients represent the change in the probability of smoking quitting for each additional effect allele.

## Supplementary Table 6. Characteristics of the genetic variants associated with heavy alcohol drinking

| **SNP** | **EA** | **NA** | **EAF** | **Chr** | **Position** | **Gene** | **Beta*** | **Se**† | **P-value** |
| --- | --- | --- | --- | --- | --- | --- | --- | --- | --- |
| rs1260326 | C | T | 0.612 | 2 | 27730940 | GCKR | 0.058 | 0.009 | 1.5E-13 |
| rs13130794 | T | C | 0.632 | 4 | 39422242 | KLB | 0.068 | 0.009 | 5.7E-16 |
| rs144198753 | C | T | 0.991 | 4 | 99713350 | BTF3P13 | 0.507 | 0.051 | 4.1E-29 |
| rs1229984 | C | T | 0.98 | 4 | 100239319 | ADH1B | 0.455 | 0.036 | 2.3E-66 |
| rs13107325 | C | T | 0.928 | 4 | 103188709 | SLC39A8 | 0.111 | 0.020 | 6.7E-09 |
| rs11214609 | G | C | 0.395 | 11 | 113316102 | DRD2 | 0.058 | 0.009 | 4.3E-09 |

Chr, chromosome; EA, effect allele; EAF, effect allele frequency; NA, non-effect allele; SE, standard error; SNP, single nucleotide polymorphism.

*The beta coefficients represent the change in the probability of being a heavy alcohol drinker for each additional effect allele.

†Part of data was inferred from the odd ratio and its 95% confidence intervals.

# Supplementary Table 7. Summary of atrial fibrillation cases and referents by ancestry

| **Ancestry** | **Atrial fibrillation case** | **Referents** |
| --- | --- | --- |
| European | 55,114 | 482,295 |
| Japanese | 8,180 | 28,612 |
| African American | 1,307 | 7,660 |
| Brazilian | 568 | 1,096 |
| Hispanic | 277 | 3,081 |
| **Total** | **65,446** | **522,744** |

## Supplementary Figure 1. Diagram of the Mendelian randomization assumptions underpinning a Mendelian randomization analysis of the association of smoking and alcohol use on atrial fibrillation


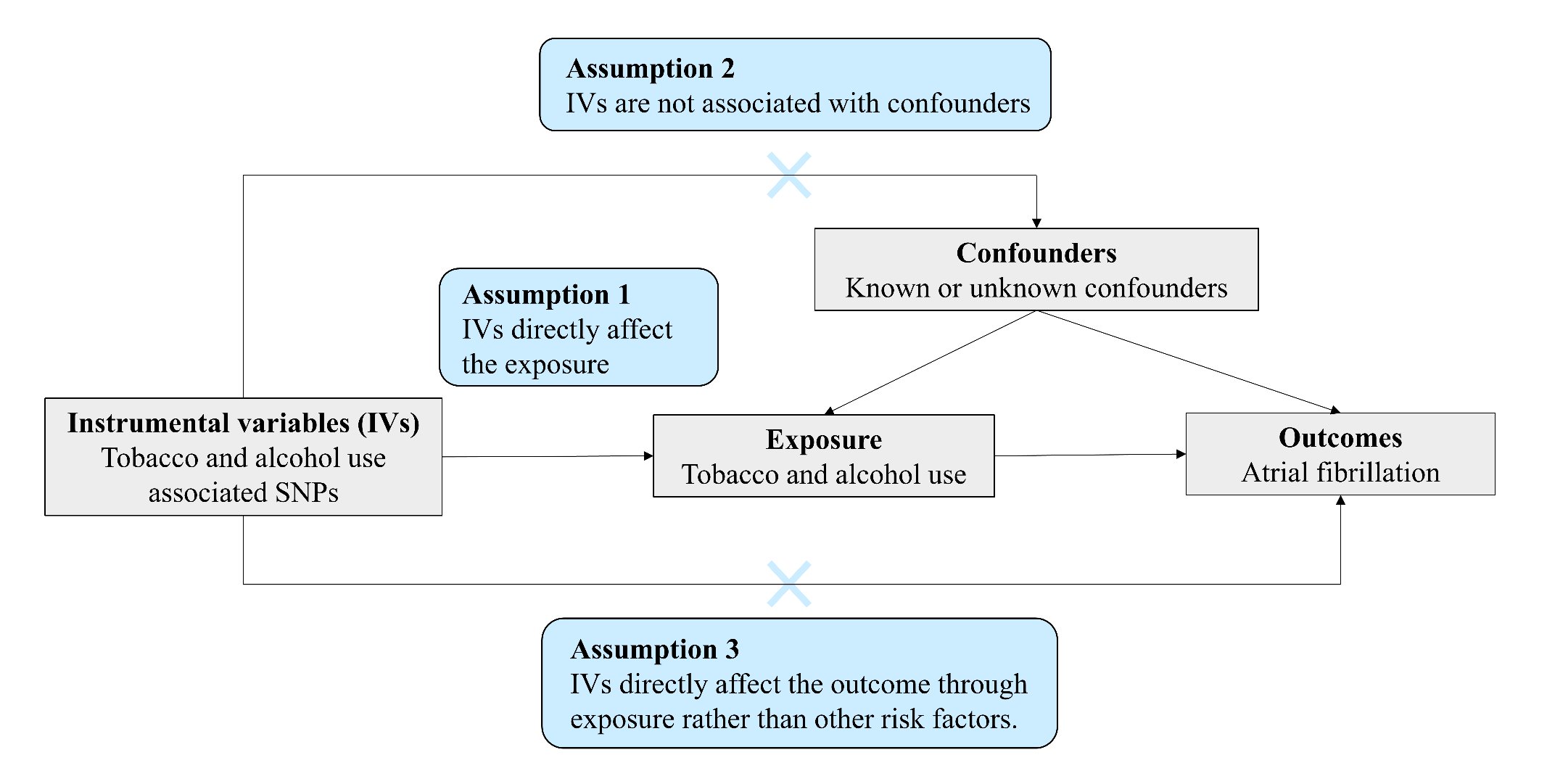


# Supplement Figure 2. Mendelian randomization association of genetically predicted smoking and heavy alcohol use with atrial fibrillation, using a linkage disequilibrium threshold of *r^2^*<0.1


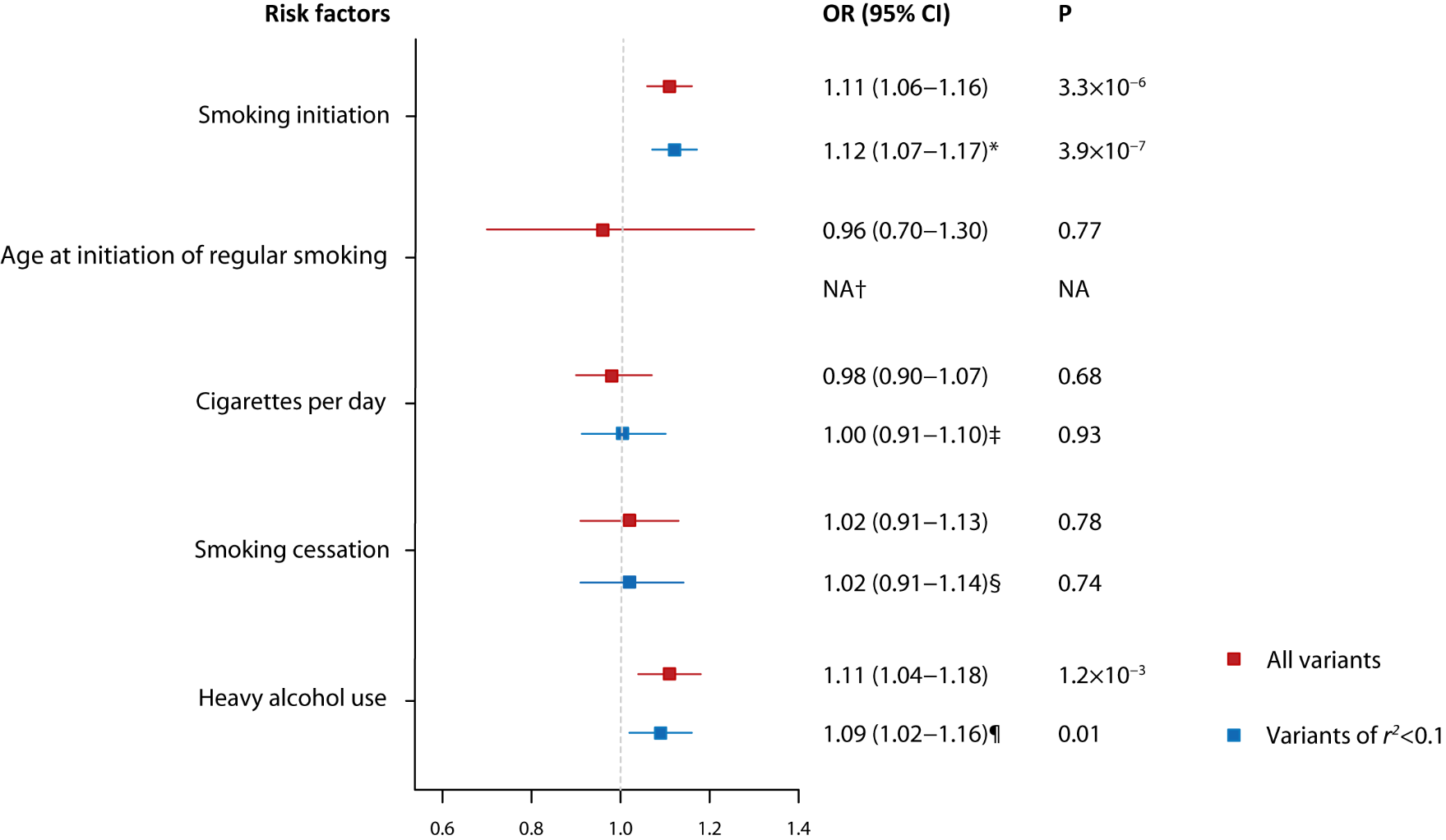


The results are derived from the fixed-effects inverse variance weighted meta-analysis.

*Instrumental variables pruned of *r^2^*>0.1: rs80054503, rs3076896, rs2196356, rs74664784, rs55900829, rs6932350, rs79180767, rs10259715, rs79631993, rs11780471, rs1565735, rs111842178, rs78239456, rs1435741, rs61537885, rs72836318, and rs6058782.

†No instrumental variables of *r^2^*>0.1 detected.

‡Instrumental variables pruned of *r^2^*>0.1: rs3025327. rs12438181, rs8040868, rs28438420, rs72740955, rs146009840, rs28681284, rs8040868, rs3743063, and rs8192726.

§Instrumental variables pruned of *r^2^*>0.1: rs59586387 and rs4809543.

¶Instrumental variable pruned of *r^2^*>0.1: rs144198753.

CI, confidence interval; OR, odds ratio.

# Supplement Figure 3. Mendelian randomization association of genetically predicted smoking and heavy alcohol use with atrial fibrillation in multiple ancestry and European ancestry


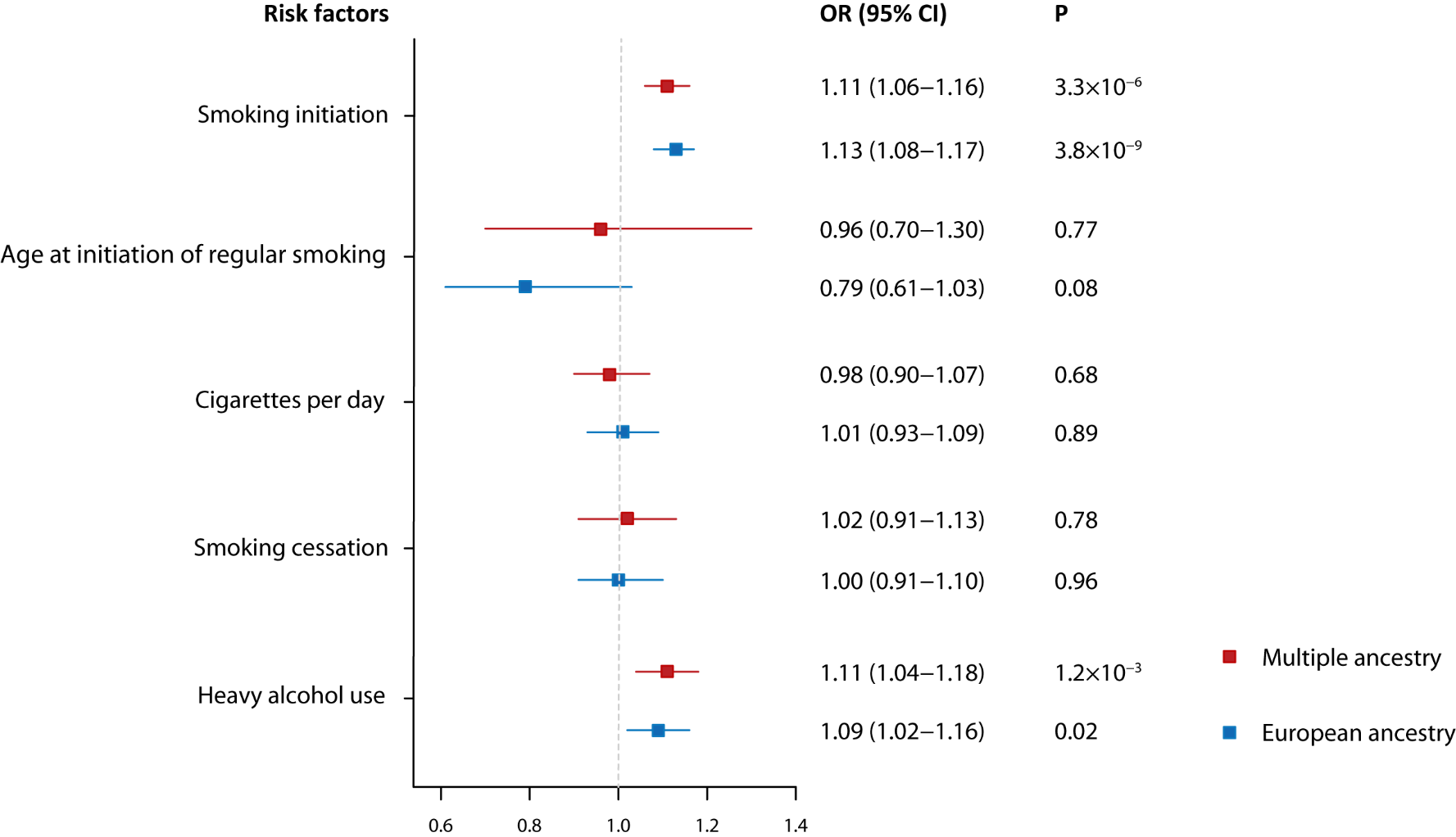


Shown are the results derived from the fixed-effects inverse variance weighted meta-analysis. CI, confidence interval; OR, odds ratio.

# Reference

1. Thompson A, Cook J. Functional validity, role, and implications of heavy alcohol consumption genetic loci. Jan 2020;6(3):eaay5034.

2. Liu M, Jiang Y, Wedow R. Association studies of up to 1.2 million individuals yield new insights into the genetic etiology of tobacco and alcohol use. Feb 2019;51(2):237-244.

3. Harris KM, Halpern CT, Haberstick BC, Smolen A. The National Longitudinal Study of Adolescent Health (Add Health) sibling pairs data. *Twin Res Hum Genet.* Feb 2013;16(1):391-398.

4. Sonnega A, Faul JD, Ofstedal MB, Langa KM, Phillips JW, Weir DR. Cohort Profile: the Health and Retirement Study (HRS). *Int J Epidemiol.* Apr 2014;43(2):576-585.
